# Manipulation of artificial and living small objects by light driven diffusioosmotic flow

**DOI:** 10.1038/s41598-024-69001-6

**Published:** 2024-08-07

**Authors:** Valeriia Muraveva, Nino Lomadze, Yulia D. Gordievskaya, Philipp Ortner, Carsten Beta, Svetlana Santer

**Affiliations:** Institute of Physics and Astronomy, University of Potsdam, 14476 Potsdam, Germany

**Keywords:** Biophysics, Optics and photonics, Physics

## Abstract

Here we report on light-triggered generation of local flow utilizing a bio-compatible non-ionic photo-active surfactant. The mechanism is based on diffusioosmotic phenomenon, where the gradient of relative concentration with respect to different chemical species near a surface leads to an osmotic pressure gradient driving liquid flow along the surface. The application of a photo-responsive surfactant allows for easy and reversible changes in concentration gradient by positioning a light source at the desired place. Along with the so-inscribed concentration gradient one can change reversible the direction and strength of the flow even in a closed system. The phenomenology of light-driven diffusioosmotic flow (LDDO) can be used in a rather flexible way: colloids can be gathered or dispersed and bio-compatibility extends the range of colloid types also to living microorganisms such as soil bacterium *Pseudomonas putida*. We show that DO flow can be considered a versatile method to set hydrodynamic conditions along the sample for investigating the motility of living cells. Further advantages of employing LDDO are the flexibility of flow generation in a reversible way and with spatiotemporal control, without the need to either change the channel geometry by loading a different device, or the periphery of pumps and connectors.

## Introduction

The natural way to set a liquid in motion is to apply an external pressure gradient, which is utilized across many length scales from industrial to microfluidics. For the latter, the plethora of electrophoretic or electroosmotic phenomena has offered viable alternatives, but the handling of electric fields that have to be applied locally remains challenging. During the last couple of years, alternative ways for inducing liquid flow have emerged employing the so-called diffusioosmosis and have made their way into the development of microfluidic-based applications^1–6^. Using diffusioosmotic phenomenon one can potentially solve many difficulties in manipulating small sample volumes (micro-liter scale) with enhanced power-consumption efficiency, simplifying the handling of fully automated systems. In the case of diffusioosmotic (DO) flow, the body force on a liquid is generated by an osmotic pressure gradient resulting from a chemical gradient of solute near a solid/liquid interface^7–9^. This effect has been known and analyzed theoretically for many years^10–15^, but was lacking a flexible practical implementation. In the early approaches the solute concentration gradients were generated globally by utilizing a rather bold microfluidic setup, where an analyte channel is connected from opposite sides to solute and solvent reservoirs, generating the flow by external pumping and fluid mixing^16–19^. There are only very few strategies for locally generating DO flow, for example based on ion-exchange reactions in polymeric structures such as ion exchange resin (IEX)^20–24^ and ion-exchanger Nafion^25^. The other option to create chemical gradient leading to DO flow involves small catalytic objects such as titanium dioxide colloids^26–30^ or natural enzymes^31,32^.

Recently we have devised a method how to induce concentration gradients to adjust the direction of DO flow and its strength on demand in a closed system. Our approach is termed light driven diffusioosmosis (LDDO) and utilizes a photosensitive azobenzene-containing surfactant that photo-isomerizes between *trans-* and *cis*-states under irradiation with light of specific wavelength^33–36^. In the case of modulated light, the photo-isomerization pattern mimics the intensity distribution resulting in an emerging concentration gradient of isomers. Since more hydrophobic *trans*- and more hydrophilic *cis*-isomers interact differently with the surface^37,38^, the concentration gradient generates an osmotic pressure gradient tangent to the wall, actuating the surrounding liquid to flow^39,40^. The direction, velocity and duration of the LDDO flow can be controlled by adjusting the wavelength and distribution of light intensity. Additionally, the LDDO approach required low light power less than 1 mW that is potentially less harmful for the living objects or sensitive materials. At this power range without the presence of the photo-sensitive substance, the particle assembly does not happen, excluding local heating or optical pressure as a possible assembling mechanism. Our approach also provides a means of implementing *virtual microfluidics* where the channels for liquid transport can be formed and shaped using optical stimuli with precise local control, in a reversible manner and at an arbitrary surface without making permanent changes to the system. Utilizing this mechanism one can manipulate an ensemble of colloids either passively, when they are driven along the DO flow, or actively in the case when the particles themselves can generate a concentration gradient^41,42^. In the latter case, small objects (for example silica colloids having pores of nano-meter size) as well as rough surfaces such as wood, dust, soil, sand, hair^43^, and microgels^44^ capable of absorbing one type of isomers (e.g. *trans*) and releasing the other one (*cis-*) can establish a permanent osmotic gradient around them and thus act as a micropump promoting a continuous flow patterns^45^. When viewed in a suitable cross-sectional plane, the flow appears as radially directed away from or towards the particles, depending on the wavelength. It is this resulting hydrodynamic flow pattern that establishes a mutual mechanical interaction between the particles which can be attractive as well as repulsive and exceeds the size of particles up to several times of their diameter^46^.

One immediately ponders whether the plethora of LDDO phenomenology can be transferred to cases where also living organisms can be manipulated as well. For this, however, the decisive prerequisite is the biocompatibility of the surfactant. In some previous studies of swimming bacteria, a non-ionic surfactant environment was used successfully for living cells^47,48^. In this work we show indeed that a non-ionic photo-active surfactant can be utilized in order to generate DO flow, which can be used to impact active swimmers and/or synthetic particles.

## Results and discussion

### Photo-isomerization of non-ionic azobenzene containing surfactant (AzoPEG)

**Figure 1:**
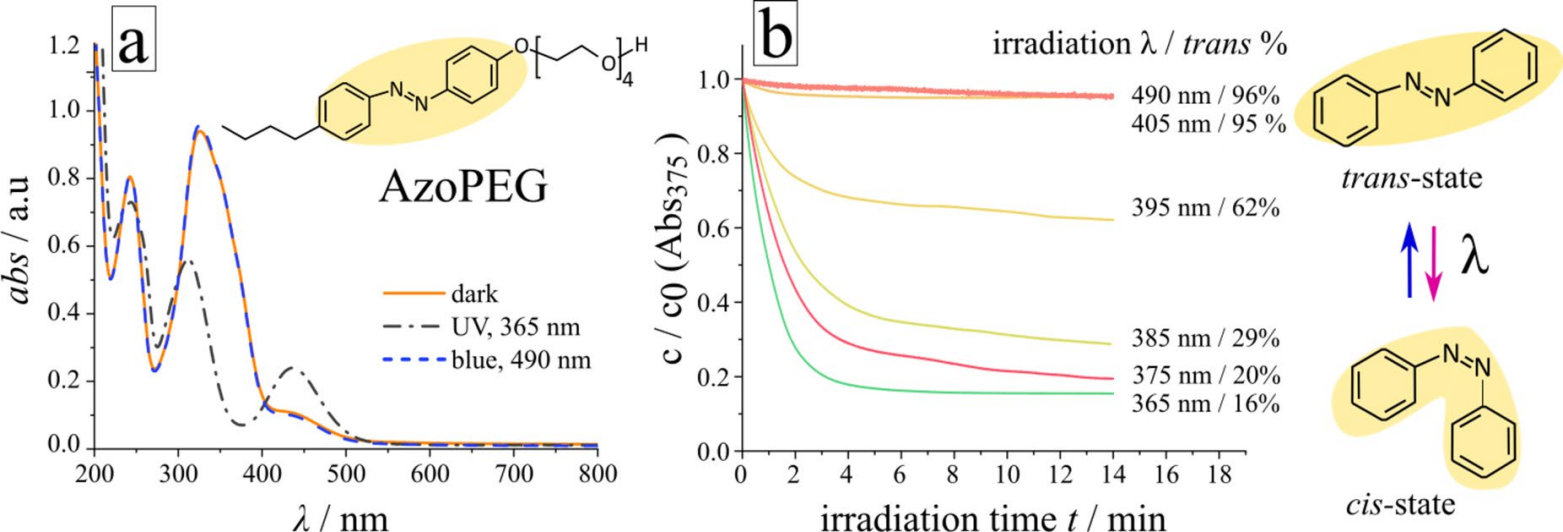
Reversible photo-isomerization of non-ionic surfactant. (**a**) UV–Vis spectra of AzoPEG surfactant solution (c = 75 µM, T = 25 °C) at photo-stationary state: under UV irradiation (λ = 365 nm, dashed-dotted black line) and under blue light (λ = 490 nm, blue dashed line), as well as after relaxation in the dark over 2 days (orange line). Chemical structure of AzoPEG surfactant is inserted. (**b**) The change in the concentration of the *trans-* isomer with time for different irradiation wavelengths between λ = 365 nm and λ = 490 nm, the irradiation intensity is I = 1 mW/cm^2^ for all wavelengths. On the right-hand side, a scheme of the photo-isomerization between *trans-* and *cis-*states is shown.

The surfactant consists of a polar tetra-ethylene–glycol headgroup coupled through an ether linkage to an azobenzene photosensitive unit with butyl tail, as illustrated in Fig. 1a. It was synthesized as described in Section 1, Supplementary Information. The absorption spectra of the surfactant in dark (initial state, the azobenzene is predominantly in the *trans* state) and under irradiation with UV and blue light are shown in Fig. 1a. The aqueous solution of *trans*-isomers (orange line) is characterized by an absorption band (π–π* transition) with a maximum at 328 nm, while the UV–Vis spectrum after irradiation with UV-light (λ = 365 nm) during 10 min, where a steady state is reached, exhibits two absorption bands with maxima at 312 nm (π–π* transition) and at 437 nm (*n*–π* transition). The band at 240 nm corresponds to the absorption of the π-conjugated benzene rings present in both isomers. Since the lifetime of the *cis* isomer in dark is 48 h, the spectra do not change considerably after the irradiation is turned off. However, when the solution is exposed to irradiation with longer wavelength, photo-isomerization from the *cis-* to the *trans-*state takes place resulting in a *trans*-isomer enriched solution (see Section 2, Fig. S2b, Supplementary Information).

The ratio of *trans* and *cis* isomers at steady state can be adjusted by using irradiation with different wavelength. Figure 1b demonstrates the kinetics of *trans–cis* isomerization as well as the ratio of *trans–cis* isomers for different wavelengths in the range between 365 and 490 nm. The decrease in the *trans*-isomer concentration is calculated by comparison of spectra of initial dark state and irradiated sample at 375 nm. Although a residual amount of *trans*-isomer may be present at this wavelength, the difference in absorptions of both isomers is maximal and the absorption at this wavelength can be assigned to the fraction of the remaining *trans* form after irradiation^49^. The amount of *trans* isomer decreases from 96% after irradiation with 490 nm to 16% after exposure to 365 nm, as shown in Fig. 1b. The *trans–cis* isomerization constant (c_azo_ = 75 µM) under irradiation with 375 nm and during exposure to 490 nm is *k*_*TC*_ = 8.16 10^–3^ and 5.17 10^–5^ cm^2^/(mW/s), respectively, and the system becomes stationary (more than 90% of molecules transformed) at 3 min for UV light and around 10 min for blue light (Fig. 1b and Figs. S2, S3 in Section 2, Supplementary Information).

### Generation of light-driven diffusioosmotic flow (LDDO)

**Figure 2:**
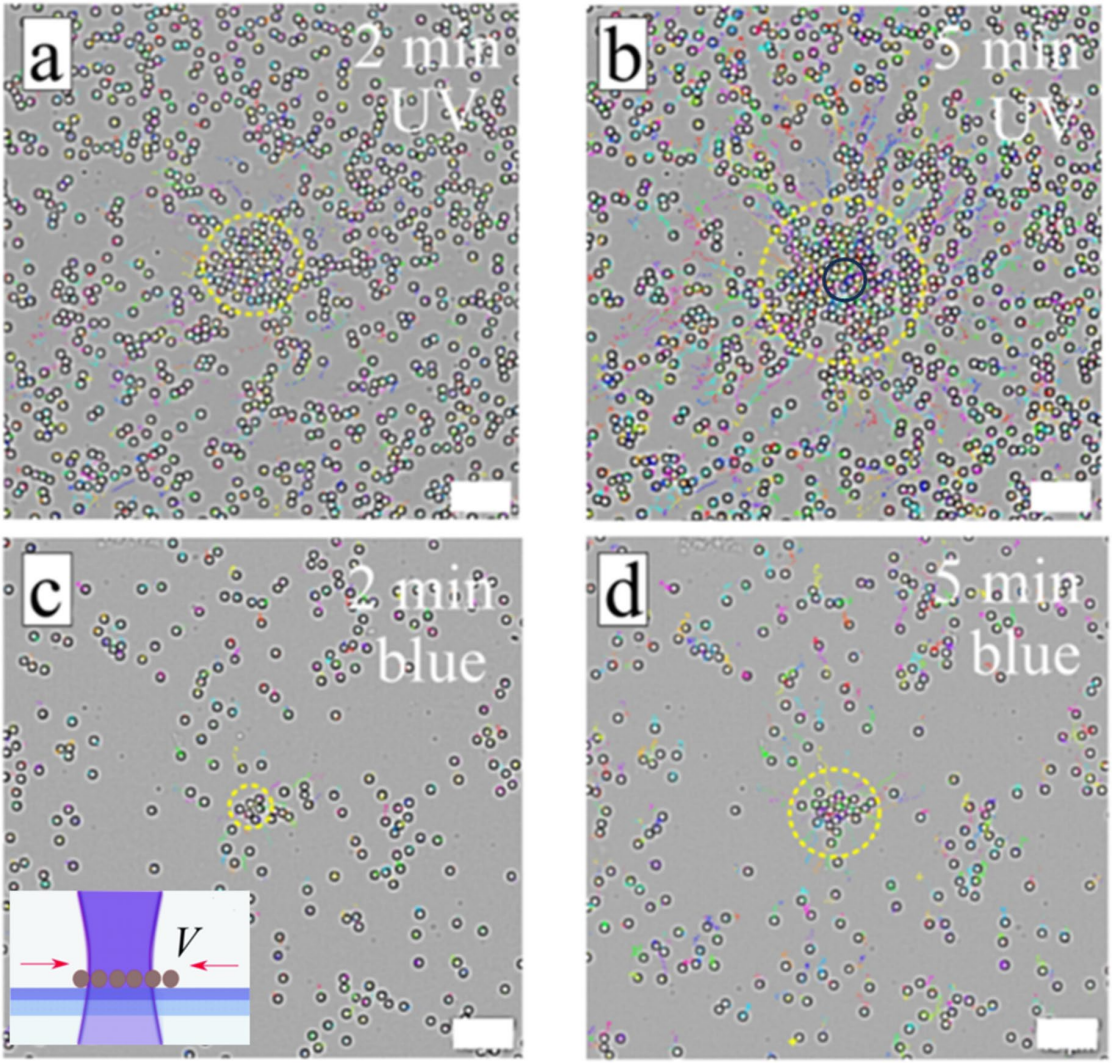
The inwards flow generated by local irradiation. Optical micrographs of the colloids (d = 5 µm) dispersed in AzoPEG (c = 75 µM) aqueous solution and exposed to irradiation with (**a**, **b**) UV (λ = 375 nm, P = 1.3 µW) and (**c**, **d**) blue (λ = 488 nm, P = 9.3 µW) light. The corresponding irradiation time is depicted on the micrographs. The trajectories of the particles representing the persistent particle displacement toward the laser spot are shown with lines of different color. Dashed circle marks the area of collected particles. The laser irradiation area, marked with black circle (*R *_*laser*_ = *15* µm) in (**b**), is constant for all experiments. Scheme of the process under irradiation is shown as insert in (**c**). The corresponding videos are provided in Video S1 and S2 (Supplementary Information).

**Figure 3:**
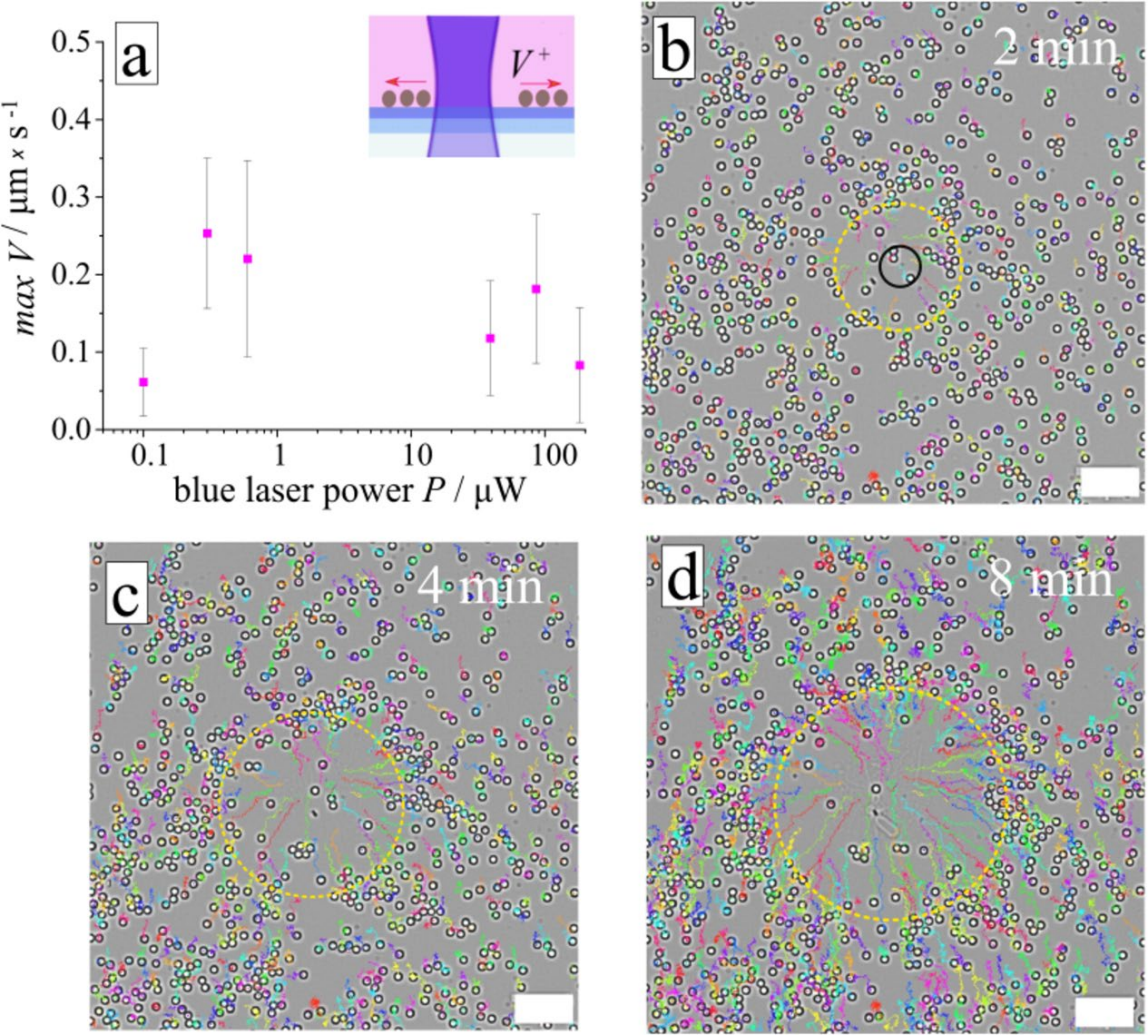
The outwards flow generated by local irradiation. (**a**) Dependence of the particles’ (d = 5 µm) average velocity on irradiation intensity during exposure to blue light (λ = 488 nm, T = 25 °C) of the *cis*-isomer enriched solution (c_azo_ = 150 µM). To keep the *cis*-isomer concentration constant, irradiation with a UV LED (λ = 365 nm, I = 3.6 mW/cm^2^) over the whole sample is switched on. (**b**–**d**) Optical micrographs and trajectories of colloids moving outwards under irradiation with blue light (λ = 488 nm, P = 39 µW). The laser irradiation area marked with black circle *R *_*laser*_ = *15 µm*. Scale bar is 40 µm. The corresponding video is provided in Video S3 (Supplementary Information).

The LDDO is generated in the presence of photo-sensitive molecules which can be switched by light between two distinct isomers having different interaction potential with the supporting surface^40^. In this way, the non-ionic azobenzene containing surfactant can act as a molecular engine for activation of fluid flow. Indeed, by applying irradiation with either UV or blue light one can activate flow either towards the maximal intensity or away from it depending on the initial conditions (Figs. 2 and 3 and corresponding videos Video S1 and Video S2, Supplementary Information). For visualization of the LDDO flow (Section 3 in Support Information), we choose tracer particles of 5 µm in diameter that are similar in size to *P. putida* bacteria (overall length of body and flagella is ca. 5 µm). Additionally, it has been shown in our previous work that the particles’ velocity in the LDDO flow does not depend on their size (at least in the range between 0.5 and 20 µm)^39^.

Before we describe the mechanism of this process in details for non-ionic AzoPEG, we would like to convey two points related to irradiation with different wavelengths as well as temperature dependence of the DO velocity.

Since blue light also switches *trans* to *cis*-state as shown in Fig. 1b, irradiation with focused light (λ = 488 nm) of the *trans*-enriched surfactant solution (i.e. equilibrated in dark for longer time) also generates inwards directed LDDO flow as illustrated in Fig. 2c, d. The extend of the flow is, however, weaker than in the case of UV irradiation, since the amount of *cis*-isomer at the photo-stationary state in this case is smaller (4% at 490 nm vs 84% at 365 nm, at room temperature).

The direction of the flow can be inverted in a closed system when the initial state of the solution is enriched with the *cis*-isomer and local irradiation is performed at longer wavelength. Here, in a first step, global irradiation of the solution with UV light transforms the surfactant to the *cis*-state. When focused blue light is switched on, the photo-isomerization from *cis* to *trans*-state results in an increased amount of the *trans-*isomer at the irradiated area. This builds up an inverted concentration gradient of the *trans*-isomers, and thus an inverted direction of DO flow depicted in Fig. 3b. At the irradiated area, the growth of worm-like objects close to the laser spot is observed which indicates the formation of aggregates of the *trans*-isomers with a large aggregation number as shown in Video S3 (Supplementary Information) and Fig. 6c. The flow points out of the irradiated area and pushes the tracer particles away cleaning up the surface as illustrated in Fig. 3. The velocity of the flow does not change much with the irradiation intensity in this particular range of the light power (Fig. 3a).

### The influence of temperature

**Figure 4:**
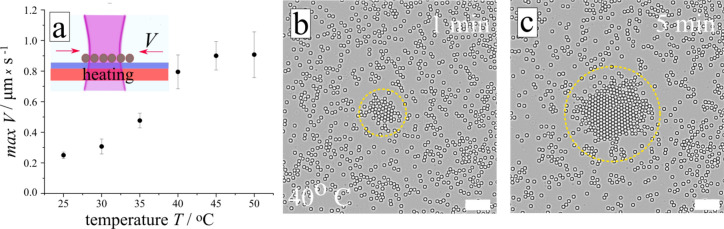
Collection of colloids by UV laser irradiation (λ = 375 nm, P = 0.7 µW) under heating. (**a**) Particles average velocity as a function of temperature, a scheme of the sample is inserted. (**b**, **c**) Micrographs of collected silica particles (d = 5 μm) at T = 40 °C, trajectories are not shown, the corresponding time of irradiation is depicted on the micrographs. Temperature is controlled by a heating stage, surfactant concentration is 75 µM. Scale bar is 40 µm. The corresponding video is provided in Video S4 (Supplementary Information).

The velocities of the particles, reflecting the LDDO flow velocity^39^, increase with solution temperature when other parameters are kept fixed (surfactant concentration, wavelength, and irradiation power). Figure 4a demonstrates the increase in the average velocity of the particles from 0.25 µm/s at room temperature to 1 µm/s at an elevated temperature of 50 °C (also see Fig. 4b, c, Section 4, Fig. S5, Supplementary Information). The increase in velocity upon heating is related to two temperature dependent processes, i.e. decrease in viscosity of water^50^ as well as re-organization of the surfactant aggregates, i.e. an increase in the amount of single molecules and/or small aggregates. The latter determines the higher gradient of osmotic pressure of solutes along the surface, while the viscosity drop yields lower friction force (see Eq. (2) and the corresponding discussion). The reorganization of the micelles from big aggregates into smaller ones is supported by the following experimental observations. The UV–Vis spectra depicts a red-shift of the absorption peak maximum of the *trans* isomer from 325 to 334 nm with increasing the temperature from 25 to 50 °C, as shown in inset in Fig. S6, Section 4 Supplementary Information, which indicates the reduction of ordered anti-parallel molecular aggregates (H-aggregates) formed by azobenzenes^51^. The transition of large elongated aggregates into smaller spherical objects upon heating is visible in optical micrographs shown in Fig. S7, Supplementary Information. The CMC of the surfactant shifts towards larger concentration with temperature as measured using the Wilhelmy plate method (Section 5, Supplementary Information) and reported early^49,52^.

With the above-described results, we state that the LDDO flow is determined mainly by the concentration gradient of single *trans*-isomer molecules and small aggregates of them. To support this further in the following, we present results of in-situ QCM measurements concerning the light driven absorption/desorption behavior of the non-ionic photosensitive surfactant at the glass surface.

### The surfactant adsorption on the surface

**Figure 5:**
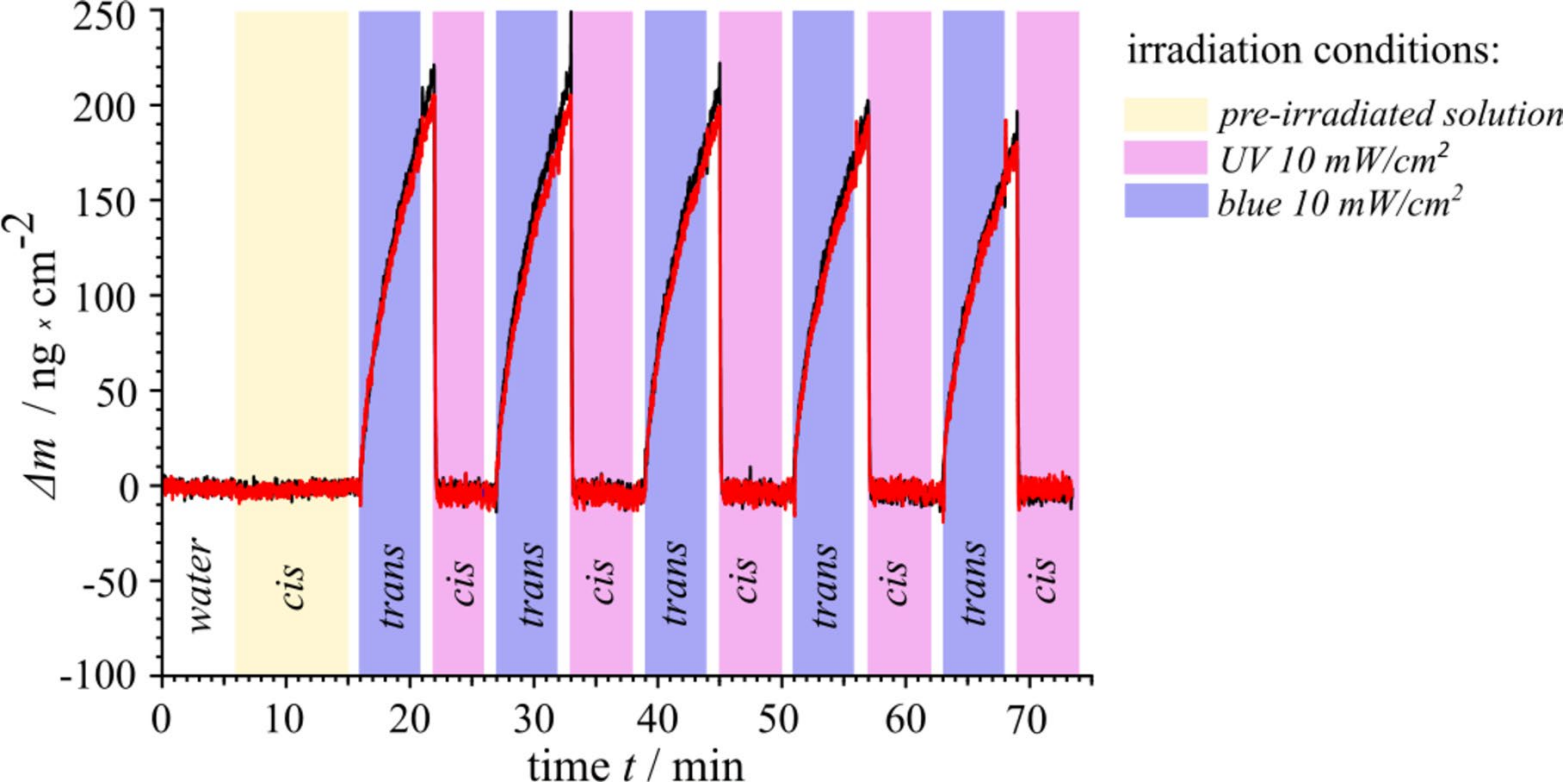
QCM measurements of the surfactant solution. The change in adsorbed mass, Δm, as a function of irradiation wavelength. The solution of AzoPEG (c = 75 µM, T = 30 °C) is pre-irradiated with UV light to convert the majority of the surfactant molecules in to *cis*-state. In the first 16 min, *cis*-isomers (yellow marked area) do not adsorb to the glass surface*.* At the areas marked in white, the pump is switched off for 1 min followed by the corresponding irradiation. As soon as the blue light (λ = 455 nm, I = 10 mW/cm^2^) is switched on, the generated *trans*-isomers adsorb to the glass surface. When the light is switched off (white area) the adsorption proceeds further, however, under UV light (λ = 365 nm, I = 10 mW/cm^2^) complete desorption is observed (violet area). The measurements are shown for two sets of experiments (black and red lines) to demonstrate the reproducibility.

The surfactant solution of a concentration of 75 µM and pre-irradiated with UV light (λ = 365 nm, P = 5mW, during 10 min) is pumped into the quartz crystal microbalance (QCM), while monitoring the change in the frequency, for details see Section 6, Fig. S9, Supplementary Information. The corresponding adsorbed mass is calculated as described in section “Methods”. As can be inferred from Fig. 5, the *cis*-isomers do not adsorb on a glass surface. After ca. 16 min of equilibration, the blue light is switched on for 4 min (transferring the majority of the molecules to the *trans*-state, indicated by the blue area in Fig. 5), which immediately results in an increase of the adsorbed mass from 0 to 200 ng/cm^2^. The subsequent exposure to UV light forces the *cis*-isomers to completely leave the surface within only a few seconds of irradiation. The process of complete desorption of the *cis*-isomers under UV irradiation and absorption of the surfactant molecules under blue light (in *trans*-state) is reversible as shown for 5 cycles of irradiation in Fig. 5. The complete desorption of the *cis*-isomers from the glass surface most probably results in low osmotic pressure, as shown by the direction of the DO flow pointing towards the irradiated area also in Fig. 2.

### Mechanism of light driven flow generation

According to Derjaguin, the DO velocity is determined by the gradient of solute concentration near the surface.^13^ In our case, as a solute one could consider both *trans-* and *cis*-isomers as well as small aggregates of them. However, since the QCM data does not show noticeable adsorption of surfactants in the *cis*-state on the surface, the contribution of *cis*-isomers can be neglected, $\frac{{\Phi_{cis} \left( z \right)}}{{k_{B} T}} \ll 1$ and the DO velocity can be expressed as follows:

1 $$\begin{aligned} v_{DO} & = - \frac{{k_{B} T}}{\eta }\mathop \smallint \limits_{0}^{\infty } z\left( {{\text{e}}^{{ - \frac{{\Phi_{trans} \left( z \right)}}{{k_{B} T}}}} - 1} \right)dz \times \nabla_{x} C_{trans}^{0} \left( x \right) = \\ & = - \frac{{k_{B} T}}{\eta }\mathop \sum \limits_{N = 1}^{\infty } \mathop \smallint \limits_{0}^{\infty } z\left( {{\text{e}}^{{ - \frac{{\Phi_{trans,N} \left( z \right)}}{{k_{B} T}}}} - 1} \right)dz \times \nabla_{x} C_{trans,N}^{0} \left( x \right) \\ \end{aligned}$$

where $C_{trans,N}^{0}$ is the bulk concentration of aggregates from N *trans*-isomers,$\nabla_{x} C_{trans,N}^{0}$ the concentration gradient of the aggregates from N *trans*-isomers along the x-axis, i.e. tangential to the surface,  $k_{B}$ the Boltzmann constant,  $T$  the temperature, $\eta$ the dynamic viscosity of the solution, $\Phi_{trans} \left( z \right)$ the potential energy of the *trans*-isomer at a distance z from the surface, and $C_{trans}^{0}$ the concentration of *trans*-isomers in the bulk.

For our particular system of the non-ionic AzoPEG surfactant under irradiation with focused UV light (i.e. *trans-* to *cis-* photo-isomerization) the DO flow is directed inwards, while in the case of a cationic photo-sensitive surfactant reported early, the direction of motion upon UV irradiation is opposite, i.e. away from the maximal intensity^40^. In the latter case, we attributed this behavior to the generation of a concentration gradient of *cis-*isomer, producing a local ion gradient adjacent to the charged surface thin electrical double layer (EDL), which in turn generates an osmotic pressure gradient pointing outward and driving a flow tangentially to the wall. The maximal velocity is achieved at a concentration larger than the CMC of *trans-* but smaller than the CMC of the *cis-i*somers of a cationic surfactant, since here the irradiation with UV light results in breaking up of micelles and release of a large number of single *cis*-molecules.

**Figure 6:**
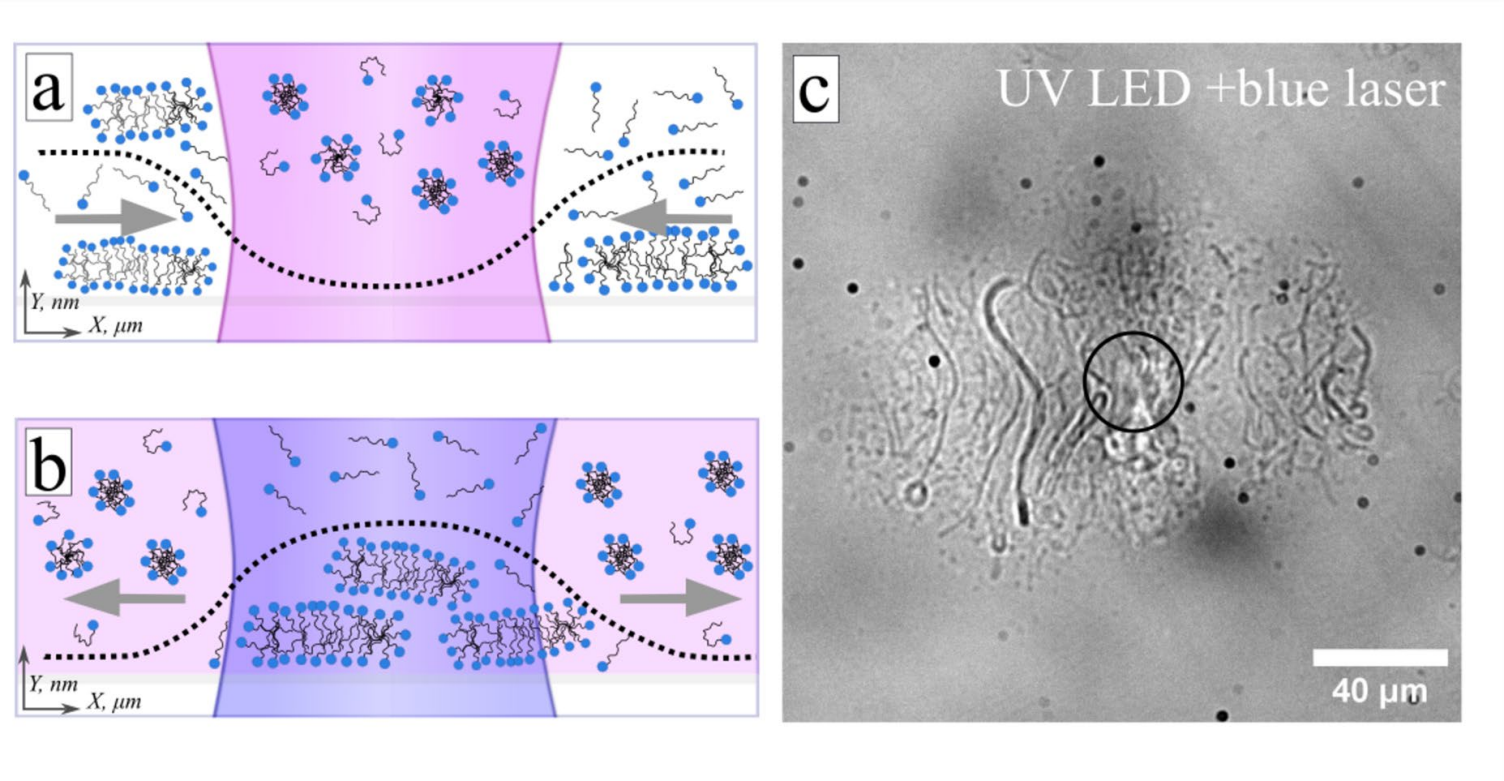
(**a**) Scheme of the system under irradiation with UV light. Worm-like micelles with larger aggregation number decomposed to spherical micelles of *cis*-isomers. (**b**) Under irradiation of *cis*-isomer enriched solution with focused blue light, the transformation of the micelles from spherical to worm-like takes place. The dotted line schematically shows the concentration of the *trans* isomer and, respectively, osmotic pressure distribution, grey arrows indicate the direction of DO flow. (**c**) Optical micrograph of the surfactant solution (c = 150 µM, prior irradiated with global UV-light (λ = 365 nm, I = 8 mW/cm^2^)) during irradiation with focused blue light (λ = 488 nm, P = 180 µW), see related sketch (**b**). Worm-like aggregates are clearly visible. The laser irradiation area marked with black circle *R *_*laser*_ = *15* µm. The corresponding Video S5 is provided in Supplementary Information.

Inwards directed LDDO flow in the solution of the non-ionic surfactant (AzoPEG) as depicted in Fig. 2a, b, implies smaller osmotic pressure of solutes where more *cis*-isomers is formed. In this case, the LDDO flow is generated at concentrations starting from ca. 25 µM (at room temperature), which is much larger than the CMC of the *trans*-isomer (CMC_rans_ = 3 µM), but comparable with the CMC of the *cis*-isomer (CMC_cis_ = 29 µM). Moreover, the value of DO velocity (starting from ca. 25 µM) does not depend on the concentration at higher c (Fig. S10, Section 6, Support Information). Such a behavior suggests that the DO flow is formed by a concentration gradient of both single molecules and small aggregates of the *trans*-isomer. Indeed, it is known that in the case of the formation of aggregates of finite size in the system, small aggregates with a maximum concentration of single molecules, $C_{trans,1}^{0} \sim CMC,$ coexist with aggregates whose aggregation number is close to the micellar aggregation number M determined by the energy gain of bond formation − α$k_{B} T, M\sim \sqrt {Ce^{\alpha } }$ (C is the total concentration of molecules)^53^. The concentration of large aggregates decreases exponentially with an increase in N, $C_{trans,N }^{0} \sim const \times e^{ - N/M}$ for N > M. Since aggregates of surfactant molecules of the order of several micrometers are observed with the optical microscope (see for example Fig. 6c), these more complex structures are formed by the aggregates with an aggregation number N ~ M as the single building blocks. Therefore, the system demonstrates a self-structuring hierarchy, where at each iteration, the aggregation number of aggregates increases, while the concentration of these aggregates decreases. So, the main contribution to the diffusioosmotic flow comes from aggregates with a lower aggregation number $N < N^{\prime}$. And since large structures disaggregate with increasing temperature in the system, the concentration of small aggregates increases resulting in a DO velocity of

2 $$v_{DO} = - \frac{{k_{B} T}}{\eta }\mathop \sum \limits_{N = 1}^{{N^{\prime}}} \mathop \smallint \limits_{0}^{\infty } z\left( {{\text{e}}^{{ - \frac{{\Phi_{trans,N} \left( z \right)}}{{k_{B} T}}}} - 1} \right)dz \times \nabla_{x} C_{trans,N}^{0} \left( x \right)$$

Thus, in the case of non-ionic surfactant, the DO flow is generated by a concentration gradient of single molecules and small aggregates of the *trans*-isomer, directed towards decreasing concentration of the *trans*-isomer as illustrated in Fig. 6a. The increase in the DO velocity at elevated temperatures results from increase in the CMC with the temperature and thus a larger number of single molecules as well as small aggregates as discussed above.

In the case of the irradiation with blue light of the *cis*-enriched solution, the concentration gradient of the *trans*-isomers is inverted as shown in Fig. 6b, i.e. at the irradiated area the large aggregates consisting of *trans*-isomer are formed (see also optical micrograph in Fig. 6c) and given larger affinity of the *trans*-isomers to the surface in comparison to the *cis*-isomers, the outwards DO flow is generated due to stronger osmotic pressure at the irradiated area.

With these results, we convey that the non-ionic surfactant can be used to generate local flow with spatiotemporal control and in a reversible manner.

### Living micro-swimmers in LDDO flow

Here we demonstrate the behavior of living micro-swimmers in LDDO flow. For this, we chose *Pseudomonas putida* which is a soil bacterium with a rod-shaped cell body and a tuft of helical flagella attached to one end of the body (polar flagellation) allowing the bacteria to swim as illustrated in Fig. 8c. The rich motile behavior of this microorganism is interesting and exhibits many similarities in the swimming pattern (run-reverse) and the flagellar morphology with some marine bacteria and several pathogenic species^54,55^. Thus, studying the swimming strategy of single *P. putida* cells could be a key to understand details of chemotaxis^56^, spreading of cells in complex environments^57–59^ and biofilm formation^60,61^ also in other polarly flagellated bacteria.

**Figure 7:**
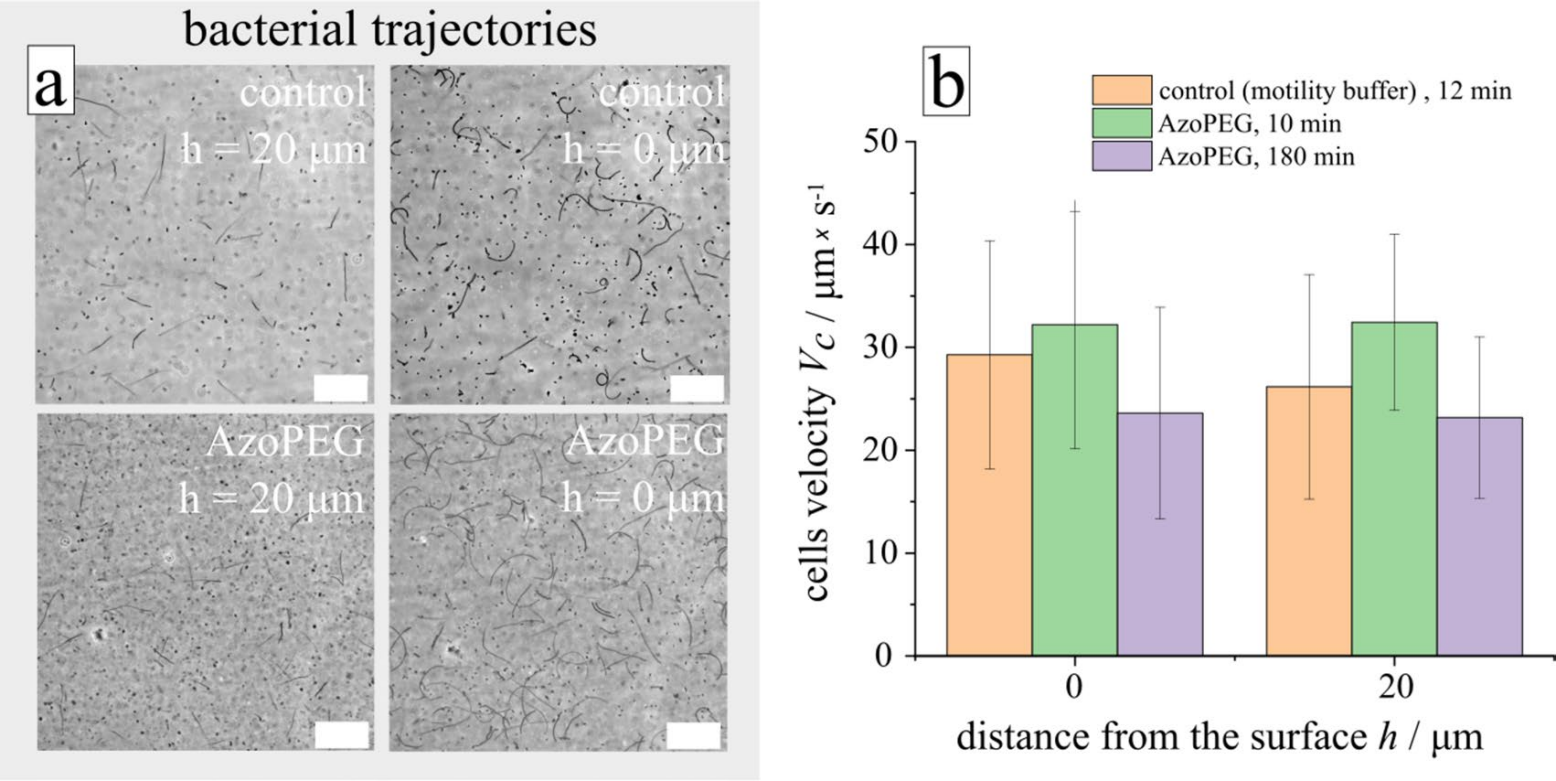
Motility of bacterial swimmers in surfactant solution. (**a**) Tracking of the trajectories of swimming *P. putida* bacteria in motility buffer (control) and in surfactant aqueous solution (AzoPEG c = 150 µM) recorded during 1 s, phase-contract imaging. The two micrographs on the left show movement above the surface at the distance of 20 µm, on the right the trajectories near the surface are shown. (**b**) Histogram of the average swimming velocities of active cells in motility buffer (control) and in non-irradiated surfactant at 15 min and 180 min after mixing bacteria and surfactant solution in bulk and close to the bottom of the chamber. Scale bar is 50 µm. Video S6 (control media) and Video S7 (AzoPEG) are provided in Supplementary Information.

First, we study the biocompatibility of the non-ionic surfactant by characterizing its impact on cell motility, the trajectories of swimming bacteria, and their velocities. As can be seen from Fig. 7a, the trajectories of the micro-swimmers close and far from the surface are similar in surfactant solution (AzoPEG) and in the case of motility buffer (control media). Also, frequencies of turns with and without photo-switchable surfactant are comparable (see Table S1, Section 7, Supplementary Information). Close to the surface in both cases, i.e. control medium and surfactant, smooth runs of cells tend to be longer than in the bulk fluid and adopt a circular shape due to hydrodynamic interactions with the wall as reported elsewhere^62–64^. The bacteria stay alive and motile during several hours in the presence and absence of the surfactant as shown in Fig. 7b. Note also that the motile properties always show variations across an ensemble of bacteria, with parts of the population showing weaker or stronger activity, i.e. some of the bacteria are moving fast with an average velocity of ca. 30 µm/s, and others are slow or even passive.

**Figure 8:**
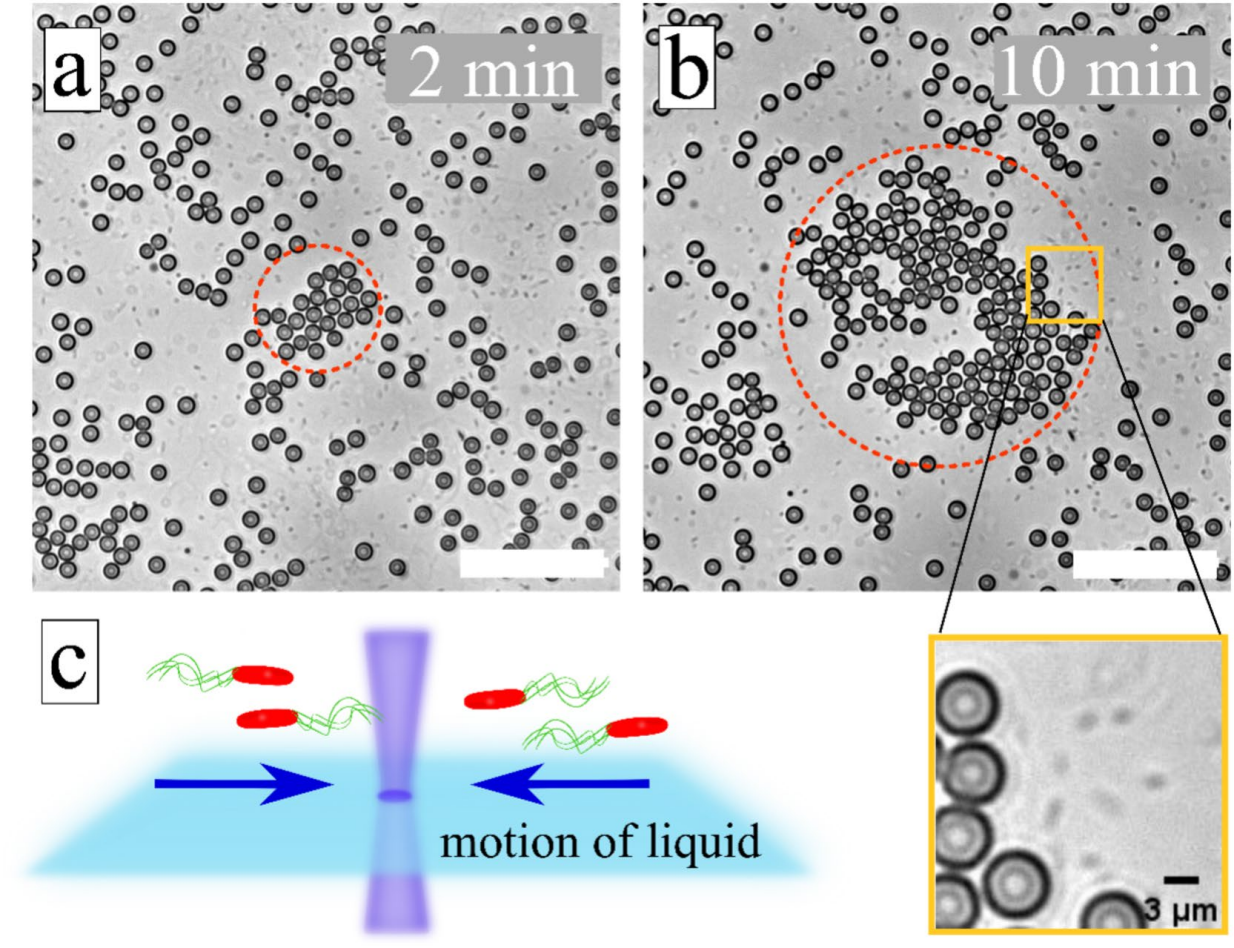
Assembling of tracer particles in the bacterial suspension. (**a**, **b**) Optical micrographs of silica particles (d = 5 µm) collected by LDDO flow during irradiation with blue laser (P = 86 µW, λ = 488 nm) at t_1_ = 2 min and t_2_ = 10 min of light exposure (red ring depicts collected particles). Holes in the collected group of particles occur due to active cell movement, which pushes particles away and destroys colloidal ordering. Small grey rod-like objects are *P. putida* cells as can be inferred from the insert in the yellow frame and from Video S8. The white scale bar is 25 µm. (**c**) Scheme of the sample: the focused laser beam at the glass-water interface induces motion of liquid and cells towards the beam center. Silica particles are not shown. The corresponding video is provided in Video S8, Supplementary Information.

To visualize the LDDO flow, we added tracer particles to the bacterial suspension (silica particles of 5 µm in diameter). When irradiation with focused blue light is switched on, the inwards directed LDDO flow advects colloidal particles as well as micro-swimmers towards the illuminated area as shown in Fig. 8 (bacterial swimmers are small black rods). The collection of less active micro-swimmers can be seen in the supplementary video (Video S8), while active micro-swimmers destroy the ordering of the colloidal assembly through a cascade of multiple collisions as can be seen in Fig. 8b and Video S8.

**Figure 9:**
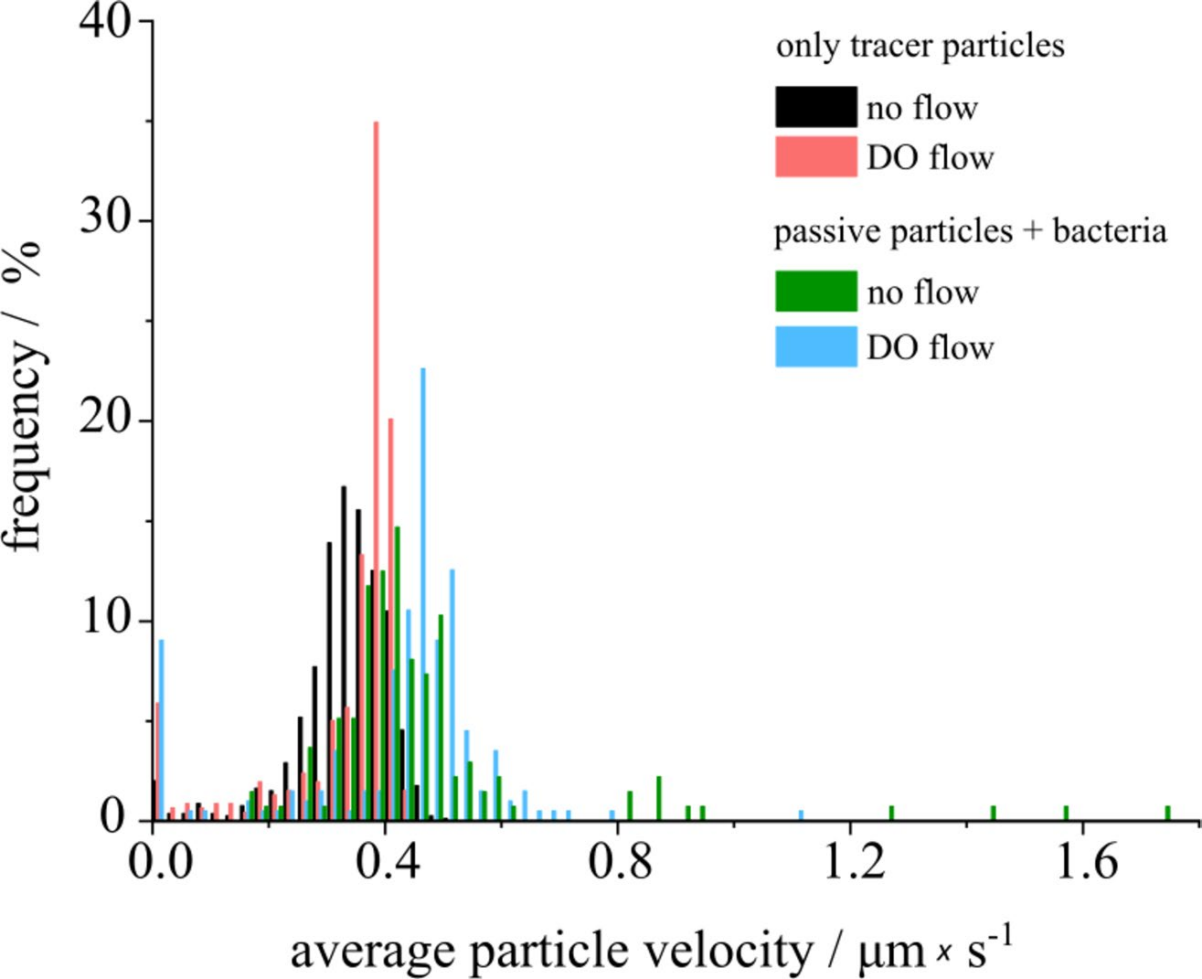
Distributions of the average tracers’ velocities along their trajectories in a dark state solution, *no flow* (black histogram) and during irradiation with blue laser (*λ* = 488 nm, *P* = 86 µW), *DO flow* (red histogram). Distributions of the velocities of tracers in bacterial suspension for *no flow* condition (green histogram) and with *DO flow* (blue histogram)*.* For all cases temperature T = 30 °C, the surfactant concentration c = 150 µM. The relative frequency is normalized by the total number of registered tracks over time N_p_ = 790 (black), N_fp_ = 457 (red), N_s_ = 136 (green), N_fs_ = 198 (blue). The corresponding video is provided in Video S8, Supplementary Information.

To elucidate the activity of micro-swimmers in DO flow, the distributions of the average velocities of tracers (colloidal particles) along the trajectories are compared for four different samples (for details of the calculations see Section 7, Fig. S11, Supplementary Information): without putida (1) in the dark and (2) under exposure to blue light, as well as in the presence of swimmers without (3) and with DO flow (4) as illustrated in Fig. 9.

In the histogram (see Fig. 9) it is shown that the tracers’ average velocities (0.3 µm/s in the dark and 0.4 µm/s under DO flow) are lower than in the presence of micro-swimmers (0.5 µm/s). Moreover, the interaction with active bacteria leads to the maximal particles’ velocities (up to 9 µm/s, see Section 7, Fig. S12, Supplementary Information) much larger than in absence of bacteria. The local destruction of the particle assembly (Video S8) and increasing of the average particle velocities demonstrate the influence of the living micro-swimmers on the tracers’ velocities, either due to collection by LDDO flow or due to spontaneous agglomeration during thermal motion (see Section 7, Fig. S13, Supplementary Information).

## Conclusion

We have demonstrated that LDDO flows can be generated utilizing a non-ionic bio-compatible surfactant. The direction and extent of the DO flow was found to depend on irradiation parameters such as wavelength and intensity as well as on solution concentration and temperature. It was shown that colloids can be either expelled from an irradiated area (irradiation with blue light of a *cis*-isomer enriched solution) or gathered within when irradiated with UV or blue light. Moreover, the behavior of *P. putida* bacterial micro-swimmers in LDDO flow in the presence of colloids was analyzed. It was found that passive and less active micro-swimmers can be collected by the flow, while active micro-swimmers (velocities of ca. 20 µm/s) destroy the ordering of the colloidal particles assembled across the irradiated area. With this paper, we lay down the groundwork for further studies regarding the mechanism of interaction of micro-swimmers with colloids with and without LDDO flow, which potentially could have an important impact on our understanding of the phase behavior of an ensemble of active and passive objects interacting in an inhomogeneous environment.

## Methods

### Light responsive surfactant

The tetraethylene glycol mono(4-butylazobenzene) ether AzoPEG surfactant as shown in Fig. 1a is synthesized and purified as described elsewhere^65–67^. In short, the surfactant is prepared in two steps: first, a hydroxyl azobenzene precursor is synthesized by diazotization of 4-butyl-anyline and following azo-coupling of diazonium salt with phenol. Further modification of 4-butyl-4′-hydroxyl azobenzene by etherification with tetraethyleneglycol tosylat gives desired AzoPEG. The details of the synthesis are provided inSection 1 and Fig. S1, Supplementary Information.

The critical micelle concentration is 3 µM for the *trans-* and 29 µM for the *cis*-isomers at 25 °C (see details in Section 5, Fig. S8, Supplementary Information).

All surfactant solutions are prepared freshly with deionized water (MilliQ) of 18.20 MΩ cm resistivity just before the measurements from a 500 µM stock solution. Irradiation was done either utilizing LED (as indicated in the text) to get *cis*-enriched solution, or by laser to generate LDDO flow. When irradiation was done by LED we indicate in the text intensity of irradiation, while in the case of laser the power is given. To calculate the power P from a given intensity I, the equation P = I×S is used, f.ex. Gene Frame area S utilized in the experiments is 1 cm², if the intensity I is 1 mW/cm², then P equals 1 mW.

*Silica particles* of 5 µm in diameter (Micromod Particle Technology GmbH, Germany) are used without further purification. The colloids are dispersion in an aqueous surfactant solution (particle concentration: 0.5 mg/ml). The dispersion is placed in a closed chamber consisting of two pieces of covered glass with Gene Frame spacer of 25 µl in volume, size 1 cm*1 cm, height h = 0.25 mm (Thermo Fisher Scientific Inc., USA).

### Cell culturing

Culture of bacterial swimmers *Pseudomonas putida* KT2440 FliC_S267C_ is grown in 5 ml of tryptone broth [tryptone (10 g/l; AppliChem) and NaCl (5 g/l)] overnight, being shaken at 300 rpm and 30 °C*.* The cell suspension from overnight cultures with OD_600_ ~ 0.4 is washed two times by centrifugation (1 min, 3300 g) and gently resuspended in surfactant solution or motility buffer (11.2 g/1 K_2_HPO_4_, 4.8 g/1 KH_2_PO_4_, 3.93 g/l NaCl, 0.029 g/1 EDTA and 0.5 g/1 glucose; pH 7.0) to concentration related to OD_600_ ~ 0.2^68^.

### Microscope setup

An inverted optical microscope Olympus IX73 equipped with a different light source is used for the bright-field microscopy imaging. To convert surfactant to *cis* form, the monochromatic UV M365LP1 LED (Thorlabs GmbH, Germany) is utilized for sample homogeneous pre-irradiation from above. Lasers with wavelengths of 375 nm (UV, Obis LX 50 mW, Coherent Inc., USA) and 488 nm (blue light, Cobolt 06-MLD 60 mW, Cobolt AB, Sweden) are used for irradiation of the sample from the bottom with focused light. Red LED (M625L1-C1, Thorlabs GmbH) is used for imaging as it does not affect the photo-isomerization of the azobenzene molecules. The illumination power is controlled by using an optical power meter PM100D with a sensor S170C (Thorlabs GmbH, Germany). The micrographs are taken with a CCD camera (Hamamatsu ORCA-Flash 4.0 LT, C11440-42C) with a speed of 1 frame per second for colloid tracking, and 5 or 20 frames per second for experiments with motile bacteria cells.

To achieve a better contrast between cells and surrounding media, for the experiments not requiring laser irradiation with bacteria another inverted microscope (Olympus IX71) equipped with an optic for the phase-contrast imaging and with a white LED as the light source was used.

*Tracking and velocity calculation* of tracers’ colloid particles and bacterial swimmers are performed using Matlab and Python codes developed in-house. Details are provided in Section 3, Fig. S4, Supplementary Information. In short, the optical micrographs are segmented into rings with fixed distances, the average velocity of a single tracer is calculated in each segment. In the sample under irradiation, the maximum average velocity is obtained in the ring close to the laser boarder, and this velocity is used further for discussions in the text.

*Heating stage* comprises an indium tin oxide (ITO) coated glass slide and two silver electrodes on either end. The temperature of the heating stage is tuned using the software, CALGrafix Version 3.1.0. and measured with a PT100 resistance thermometer attached to the glass side. The temperatures are adjusted between room temperature 25 °C and 50 °C.

*UV–Vis spectroscopy* measurements of kinetic are performed with a Cary 5000 UV–Vis-near-infrared (NIR) spectrophotometer equipped for temperature control and stirring (Agilent Technologies, USA) in a quartz cuvette with light pathway L = 10 mm (Hellma Group, Germany).

*Quartz crystal microbalance with dissipation* (QCM-D) measurements are performed with a four-chamber Q-Sense E4 instrument (Biolin Scientific, Sweden) using Q-Sense crystals coated with borosilicate (QS-QSX336, LOT Quantum Design GmbH, Germany). Adsorption is acquired by monitoring the frequency shift Δ*f* and the dissipation Δ*D*, from the 3rd to the 9th overtone. Each solution is introduced into the QCM chamber at a flow rate of 50 μL/min using a peristaltic pump. The adsorption of the surfactant is investigated *in-situ*, i.e. during irradiation. For this the *cis-*isomers enriched solution is injected into the QCM, the evolution of Δ*f* and Δ*D* is recorded until an equilibrium state is reached, i.e. when no further change in the observables occurs. At this point, the irradiation with light of 365 nm wavelength is switched on, where the light incidents through the specially designed window in the chamber of the QCM. Prior to each measurement, the resonance frequency of the crystal is determined first in air, then in degassed Millipore water, and under irradiation with light of appropriate wavelength. The latter is done for the light induced detuning (LID) correction^69–71^, i.e. the correction of the QCM sensor response on just irradiation^72^. In short, when measuring QCM-D and illuminating the quartz crystal with LED light source (*λ* = 365 nm, *I* = 20 mW/cm^2^) one observes a positive frequency (Δ*f*) and negative dissipation shift (Δ*D*) which maintains in a steady state during the whole illumination period. To correct it we develop a five-step procedure beginning with LID-baseline recording (I) with the same illumination parameters and the same irradiation time as during the measurements themselves (II). The normalized mass (mass/area) as a function of the time is calculated from data (I) and (II) into (III) and (IV) using modified Sauerbrey relation:^73^

3 $$m\left( t \right) = - \frac{C}{n} \cdot \left[ {\Delta f\left( t \right) + \left( {\frac{{f_{C} \cdot \Delta D\left( t \right)}}{2}} \right)} \right]$$

where mass model calculation resulting from LID is considered to be an unknown interfacial interaction assuming small load approximation. Equation (1) is used with* C* as the crystal constant for the resonance crystal frequency *f*_C_ (~ 5 MHz of company q-sense), *n* the overtone number, *m*(*t*) the normalized mass (mass/area), Δ*f*(*t*) and Δ*D*(*t*) at any time *t*. The corrected LID artifact (V) is obtained by subtracting the data (IV)–(III):

4 $$\left( V \right) m\left( t \right) = \left( {IV} \right) m_{abs + baseline} \left( t \right) - \left( {III} \right) m_{baseline} \left( t \right)$$

After each measurement, the borosilicate-crystals (QSX 336) are treated for 10 min with a plasma cleaner followed by washing in a series of cleaning solutions for 30 min: first in SDS (2 wt%) followed by Millipore water, keeping the crystal always wet prior to drying with a stream of N_2_. Subsequently, the crystals are treated again by Plasma cleaning for 10 min.

### Supplementary Information

Supplementary Information.

## Data Availability

Data is provided within the manuscript or supplementary information files.

## Code availability

The code was utilized for imaging tracking and further analysis are available from the corresponding author on reasonable request.
