# Supplementary material for: Manipulation of artificial and living small objects by light driven diffusioosmotic flow
Source: Sci Rep. 2024 Aug 7;14:18342. doi: 10.1038/s41598-024-69001-6 (PMC11306628; doi:10.1038/s41598-024-69001-6)
Supplement: Supplementary file 1 — Supplementary Information. [file 41598_2024_69001_MOESM1_ESM.zip › Edited Supplementary Information.docx]

**Supplementary Information.
Manipulation of artificial and living small objects by light driven diffusioosmotic flow**

*Valeriia Muraveva, Nino Lomadze, Yulia D. Gordievskaya, Philipp Ortner, Carsten Beta, Svetlana Santer**

Institute of Physics and Astronomy, University of Potsdam, 14476 Potsdam, Germany
E-mail: santer@uni-potsdam.de

**Section 1. Synthesis of the tetraethylene glycol mono(4-butylazobenzene) ether (AzoPEG)**

Tetraethylene glycol mono(4-butylazobenzene) ether (**AzoPEG**) is prepared in two steps: first, hydroxyl azobenzene precursor is synthesized by diazotization of 4-butyl-anyline and following azo-coupling of diazonium salt with phenol. Further modification of 4-butyl-4’-hydroxyl azobenzene by etherification with tetraethyleneglycol tosylat gives desired tetraethylene glycol mono(4-butylazobenzene).



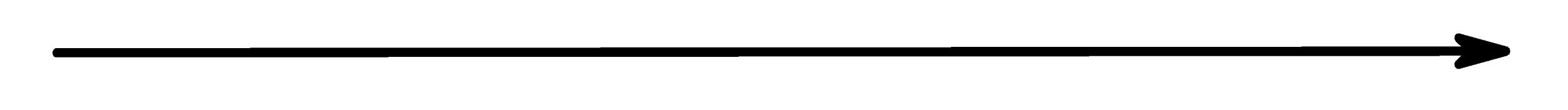


**Figure S1**. Synthesis of the photo-switchable non-ionic surfactant 2-[2-[2-[4-[2-(4-buthylphenyl)diazenyl]phenoxy]ethoxy]ethoxy]ethoxy]ethanol

The synthesis was carried out as described in the literature:^1^

Final product is characterized by ^1^H NMR:



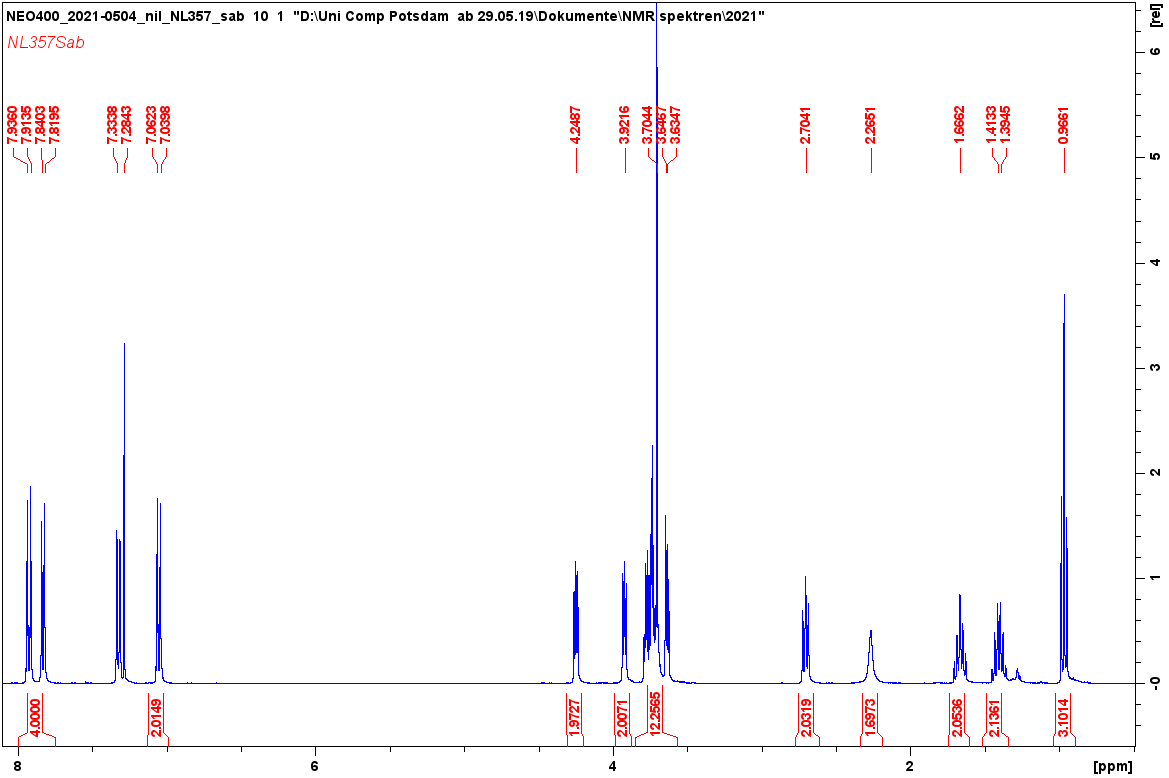


**Section 2. Photoisomerization of AzoPEG surfactant**

*
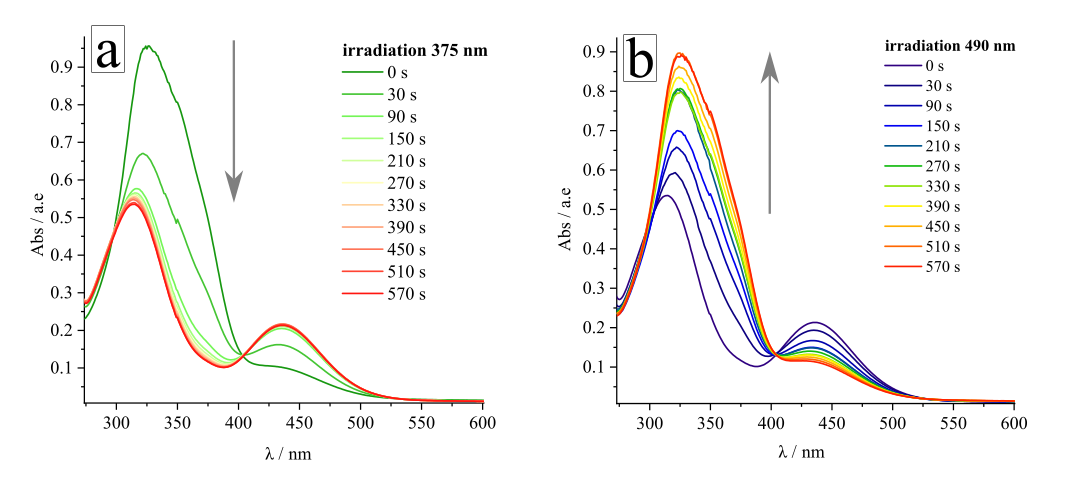
*

**Figure S2**. Kinetic of the AzoPEG (c = 75 μM, T = 25° C) photo-isomerization. (a) Changing of UV-Vis spectra showing *trans-cis* transition under illumination with UV light (λ = 375 nm, I = 1 mW/cm^2^). (b) Spectra showing transition from *cis* to *trans* form under blue light irradiation (λ = 490nm, I = 1 mW/cm^2^ )

***Calculation of isomerization constant***

The kinetic of photo-isomerization is studied by irradiation of surfactant aqueous solution (c =75µM) by different LED light sources with fixed intensity I = 1 mW/cm^2^. The decay time *τ* obtained by fitting the results from **Figure 1b** in the main text using **Equation 1**:


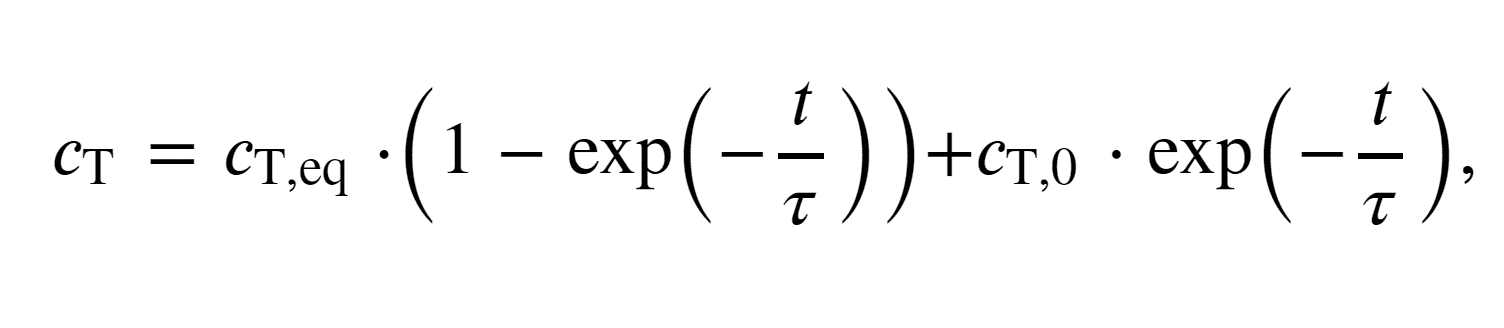
(1)

The photo-stationary state is achieved with 14 min of irradiation. The isomerization constant *k_trans-cis_* is obtained using following **Equation 2** (**Figure S2)**:


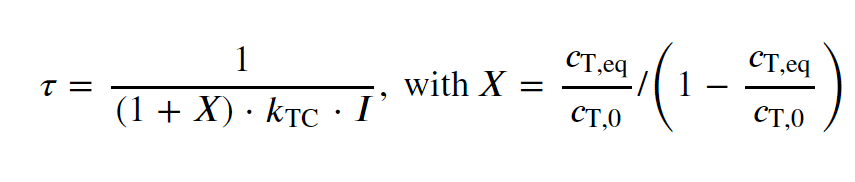
(2)


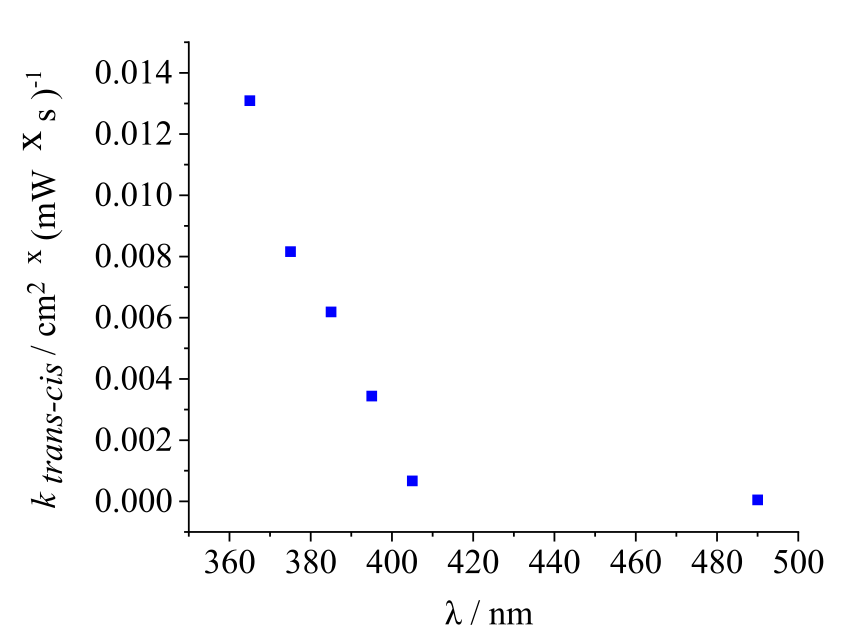


**Figure S3.** The isomerization constant (*trans-cis* conversion) is plotted as a function of irradiation wavelength. Surfactant concentration is c= 75µM, for all light sources I =1 mW/cm^2^, measurements are performed at T = 25 °C.

**Section 3. Tracking of tracer particles**

Recognition and tracking of objects are extracted utilizing the lab-made MATLAB and Python code.

*Model for colloid motion*. In order to extract the colloid speed as a function of distance from the hotspot created by the laser, we model the dynamics of colloidal tracers by a stochastic differential equation in a similar way to how was discovered earlier.^2^ Let $R$ be the position of the hotspot and $r_{i}\left( t \right)$ the trajectory of the *i-*ths particle. We describe the dynamics of colloids by

$$\dot{r}_{i}\left( t \right)=v\left( \left| r_{i}-R \right| \right)\frac{r_{i}-R}{\left| r_{i}-R \right|}+\xi_{i}\left( t \right),$$

Where the stochastic noise $\xi_{i}\left( t \right)$ satisfies the condition for fluctuations*.* $\langle\xi_{i}\left( t \right)\rangle=0$

The tracking yields $N$ sampled trajectories of colloids $r_{i}\left( t_{\mu} \right)$ where $i=1,2,\ldots,N$ indicates the particle index and $\mu=1,2,\ldots,n_{i}$ the frame number ($n_{i}$is the total number of frames in trajectory $i$). We estimate the instantaneous velocity of colloids by a finite difference scheme:

$$V_{i}\left( t_{\mu} \right)=\frac{r_{i}\left( t_{\mu+1} \right) - r_{i}\left( t_{\mu} \right)}{\Delta t} .$$

The temporal resolution is denoted by $\Delta t=1s$ or $\Delta t^{'}=0.2s$ for experiments with cells.

In order to estimate the flow-induced colloid speed $v\left( l \right)$ as a function of the distance $l=\left| r_{i}-R \right|$ to the hotspot position, we first create a histogram of these distances; the bin edges are denoted by $l_{m}$, where $m=1,2,\ldots,B+1$ enumerates the bins (total bin count: $B$). We were also interested in the time dependence of the velocity of colloids. The effective mean colloid speed at the bin centers over a period of time $\left[ t_{\mu_{1}}, t_{\mu_{2}} \right]$ can hence be estimated by averaging over all particles that are found in a specific bin at time $t_{\mu_{2}}$ during the recording

$$v\left( \frac{l_{m+1}+l_{m}}{2}, t_{\mu_{2}} \right)\approx\frac{1}{K} \sum_{i=1}^{N} \sum_{\mu=\mu_{1}}^{\mu_{2}-1} \theta\left( l_{m+1}-\left| r_{i}\left( t_{\mu} \right)-R \right| \right)\theta\left( \left| r_{i}\left( t_{\mu} \right)-R \right| {- l}_{m} \right)V_{i}\left( t_{\mu} \right)\cdot\frac{r_{i}\left( t_{\mu} \right)-R}{\left| r_{i}\left( t_{\mu} \right)-R \right|}$$

where the Heaviside $\theta$-functions ensure that the particle belongs to bin number $m$ and $K$ abbreviates the number of terms in the sum:

$$K= \sum_{i=1}^{N} \sum_{\mu=1}^{n_{i}-1} \theta\left( l_{m+1}-\left| r_{i}\left( t_{\mu} \right)-R \right| \right)\theta\left( \left| r_{i}\left( t_{\mu} \right)-R \right| {- l}_{m} \right).$$

This expression is formally an estimate for the first Kramers-Moyal of the stochastic colloid dynamics. In the main part of the paper, the flow-induced colloid speed $v\left( l \right)$ is referred to as $V_{average}\approx v\left( 10-20\mu m, 30s \right)$; specifically, we focused on the mean colloid speed close to the hotspot ($distance from the laser center l\approx0-30\mu m$) for a short time after turning on the laser. In the **Figure S3** one may see that average velocity close to the laser spot decreases and reaches zero after several minutes of the irradiation by UV-laser, it’s connected with collecting the particles in the center, at that moment they cannot move further due to steric restrictions.


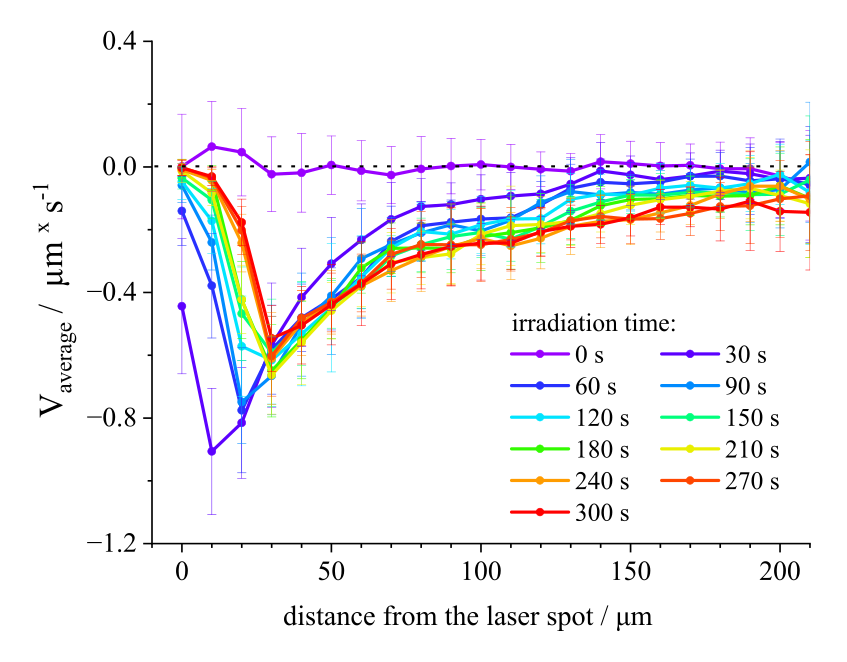


**Figure S4**. The distribution of velocities of silica particles (dimeter of particles is 5µm) as a function of the distance *l* to the laser spot (*λ* = 375 nm, *P* = 0.7 µW) at different time after laser on, azo-surfactant c = 75 µM.

**Section 4. Temperature effects on LDDO flow**


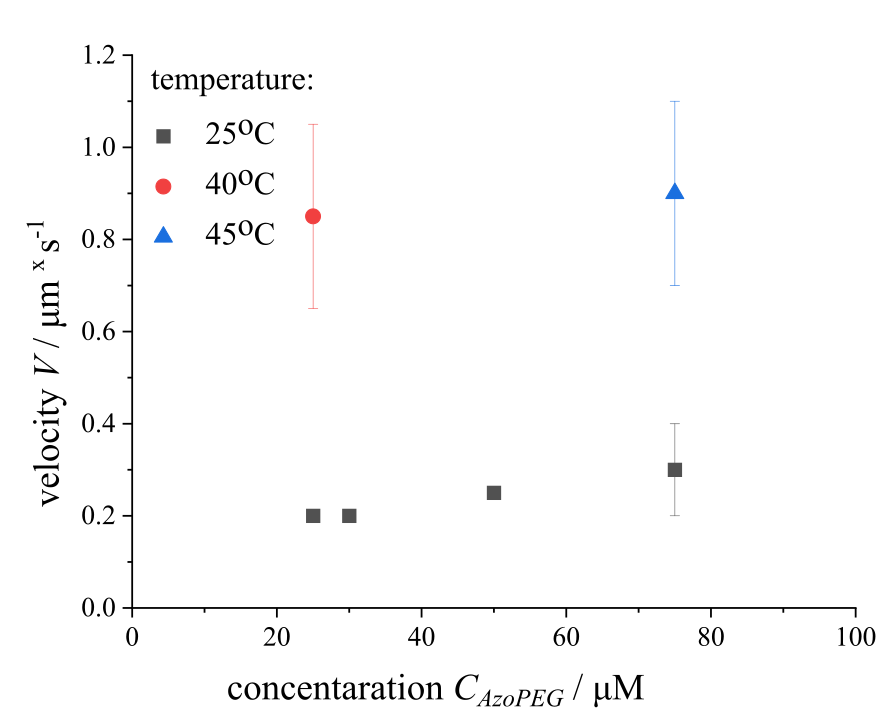


**Figure S5**. The average velocity of tracers as a function of surfactant concentration in the case of the irradiation of dark state solution with UV-laser (*λ* = 375 nm, *P* = 0.7 µW) which leads to collection of particles.


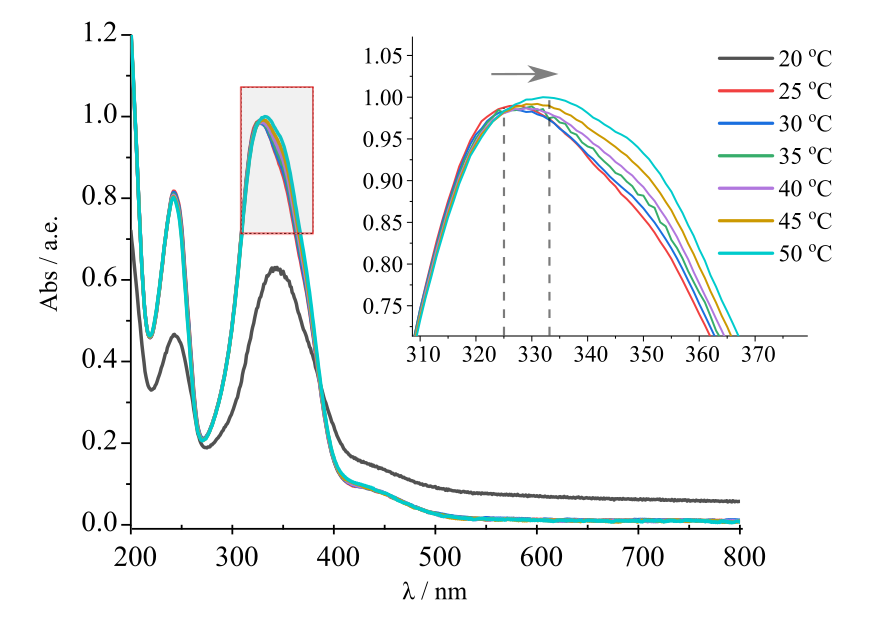


**Figure S6.** Changing of UV-Vis spectra during temperature increase. Insert depicts a shift of the peak center to the longer wavelength. Dashed lines indicate the maximum of adsorption.


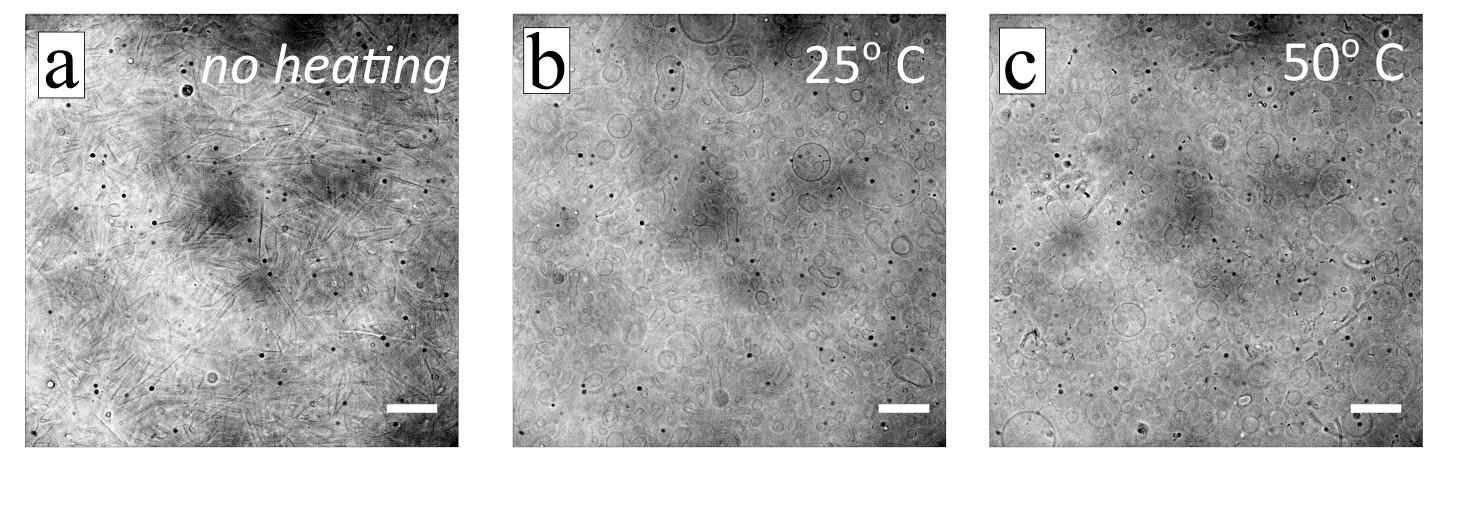


**Figure S7**. Optical micrographs (bright field microscopy) with optimized contrast showing the transition of big elongated aggregates (a-b) into smaller spherical objects(c) under heating. The surfactant concentration is c = 300 μM, heating time 5 minutes, scale bar is 40 µm.

**Section 5. Measurements of critical micelle concentration of *trans* and *cis*- isomers AzoPEG**

For critical micelle concentration (CMC) estimation we apply the Wilhelmy plate method. Surface tension data are obtained by force tensiometer K 100 equipped for temperature control (Krüss, Germany), see **Figure S7**. For *trans* isomer all experiments are performed in the dark, for *cis* isomers illumination is conducted by LED UV source (λ =365 nm) before experiment. Samples are preheated before experiments up to the target temperature during 10 minutes with additional stirring.

In order to avoid an effect of the temporal evolution of the surface tension (due to the competitive absorption of different isomers or thermo-relaxation effect), the surface force measurements are done 15 s after start of the layer formation.


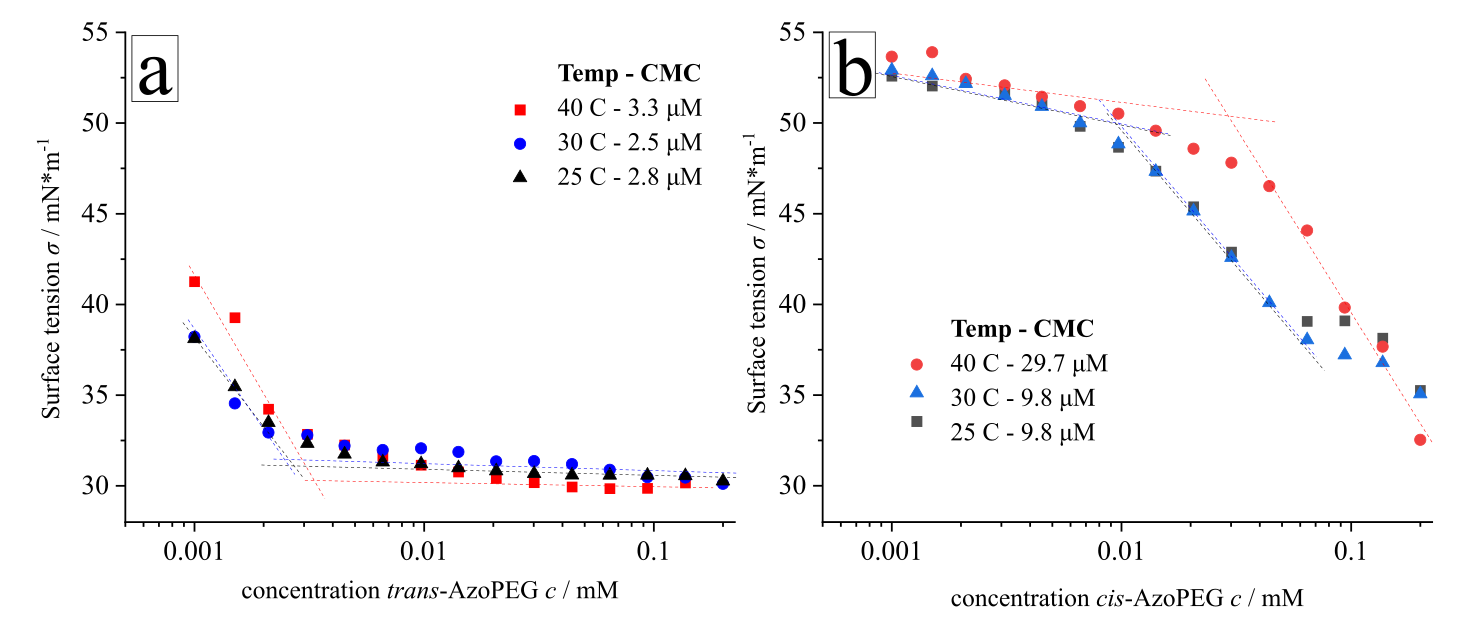


**Figure S8**. The surface tension as a function of concentration under different temperatures for (a) *trans-*AzoPEG surfactants (dark state solution) and (b) *cis*-AzoPEG (UV light pre-irradiated solution). The lines cross marked a transition and start of the aggregate formation

**Section 6. Quartz crystal microbalance (QCM) measurements of AzoPEG surfactant**

| **** | **** |
| --- | --- |

**Figure S9**. Typical results of QCM measurements during adsorption/desorption of AzoPEG surfactant on a glass surface shown for two sets of experiments (left and right) to demonstrate the reproducibility. Frequency shift, Δf, and dissipation shift, ΔD, as a function of time, t, for the overtone numbers n = 3, 5, 7, 9 (black, red, green, and blue curves, respectively). The surfactants are in 100% cis isomeric state (after irradiation with UV light), the concentration is 75µM. In the first 16 minutes of measurements (yellow marked area) the *cis*-isomers do not adsorb to the glass surface. As soon as the blue light (λ=455nm, I=10mW/cm^2^) is switched on, generated *trans*-isomers starts to adsorb to the glass. When the light is switched off (white area) the adsorption proceeds further, but under UV light (λ=365nm, I=10mW/cm^2^) complete desorption sets on (violet area). The change in mass calculated using **Eq. 1** from the main text is shown below. Using this plot **Figure 5** in the main text is generated by subtraction of the light induced detuning in QCM-D experiments (LID) signal generated by light at the sensor surface as described elsewhere.^3^

**Section 6. Concentration effect**


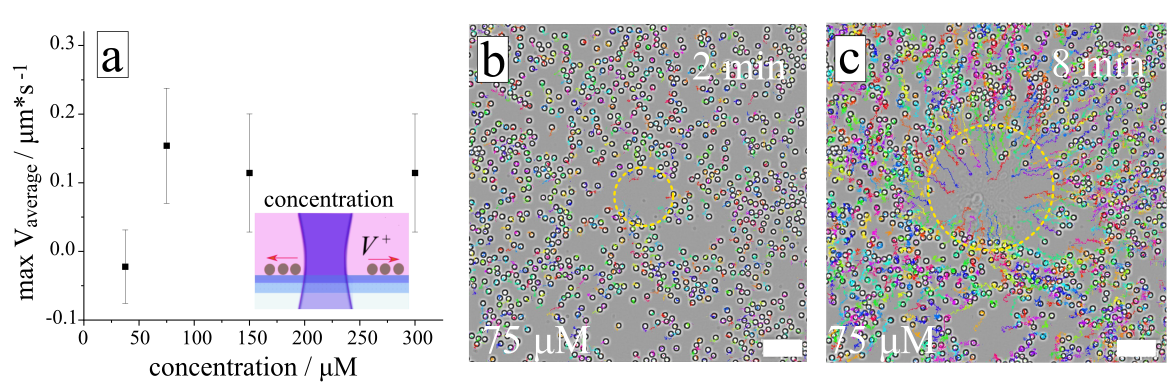


**Figure S10**.The average velocity of tracers as a function of surfactant concentration under irradiation with focused blue light (λ=488nm, P=2.9 µW) and global UV light (λ=365nm, P=11.5 mW), T=25°C. The inserted scheme illustrates the direction of particle motion relative to the maximal intensity.(b, c) Optical micrographs of the tracked colloids (d=5µm) together with their trajectories pushed away of the center of laser point in *AzoPEG* solution (c = 75 µM*).* Scale bar is 40 µm. (*One can compare it with figure 3 in main text, doubled concentration 150 µM).*

**Section 7. Bacterial swimmers in AzoPEG solution**

To study the influence of surfactant presence on the cell’s motility, trajectories of cells were tracked and analyzed. Cells velocities, length of tracks and event rate (how often bacteria switched run direction) are calculated for control media – motility buffer (mixture of salts and glucose), see **Table S1**. The same parameters were analyzed for *trans*-AzoPEG surfactant solution (c =150 μM, the solution was kept in the dark before) and *cis*-AzoPEG solution of the same concentration, but preirradiated (t=30 min, λ = 365 nm, I = 1 mW/cm^2^) before mixing with bacteria suspension for conversion of the molecules to the *cis*-form.

The recorders are done with a frame rate of 20 frames per second by phase contrast microscopy. For example, please see Supplementary **Video S6 and Video S7.**

| **media** | **time / min** | **distance from the bottom / µm** | **mean track length, µm** | **event rate / s^-1^** |
| --- | --- | --- | --- | --- |
| motility buffer  (control) | 12 | 0 | 30.43 | 0.677 |
|  |  | 20 | 20.09 | 0.412 |
| *trans*-AzoPEG  (c=150 µM) | 10 | 0 | 28.95 | 0.163 |
|  |  | 20 | 14.11 | 0.106 |
|  | 180 | 0 | 26.32 | 0.574 |
|  |  | 20 | 17.65 | 0.595 |
| *cis*-AzoPEG  (c=150 µM) | 17 | 0 | 25.67 | 0.631 |
|  |  | 20 | 19.80 | 0.554 |
|  | 189 | 0 | 30.76 | 0.765 |
|  |  | 20 | 25.08 | 0.619 |

**Tabel S1.** Frequencies of events (turns), track lengths, run time (time between two turs) for bacterial swimmers with and without photo-switchable surfactant.

Noticeable, that the event rate for the *trans*-surfactant is much height in comparison with control media and *cis*-surfactant at the beginning of measurements, the runs interrupted by turns more frequent. This is related to the fact, that *trans*-surfactant absorbs on the surface and forms big aggregates which play the role of “soft confinement”. It is supported by the QCM measurements of absorbing surfactant mass (see **Section 2.4** in **Main Text**) and micrograph of aggregates (**Figure 6, Main Text**). With time, the cells' activity may destroy aggregates or aggregates get small due to microscope lamp irradiation (leads to transition to the *cis-*isomer with larger CMC) during the measurements.

***Movement of tracer particles in AzoPEG solution in the absence/presence of swimmers***

We found that bacteria interact with particles in a manner that leads to the particles exhibiting significant displacements, or the same as having high instantaneous velocities. To provide a clear illustration of our observation, we chose to comprehensively examine the entire path of each particle throughout the observation period. As a particle is more frequently affected by bacteria, it results in more large displacements along its path and subsequently raises the average speed of the particles along their trajectories (we should pick up the name for this value):

$$v_{i}=\frac{\sum_{\mu=1}^{\mu=n_{i}} \left[ r_{i}\left( t_{\mu+1} \right) - r_{i}\left( t_{\mu} \right) \right]}{\sum_{\mu=1}^{\mu=n_{i}} \Delta t_{\mu}}$$

where $n_{i}$ is a total number of frames for i-particle and $\left[ r_{i}\left( t_{\mu+1} \right) - r_{i}\left( t_{\mu} \right) \right]$ shows the displacement over a frame $\Delta t_{\mu}$.


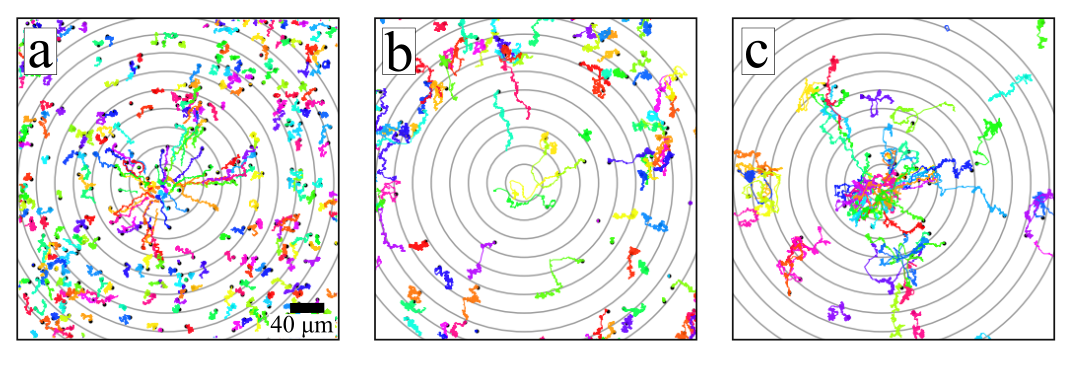


**Figure S11**. Trajectories of all tracers (a) in AzoPEG solution (150µM, 30° C, *λ* = 488 nm, *P* =86 µW). Trajectories of tracers with an average velocity along the trajectory less than 0.5µm/s (b) and greater than 0.5µm/s (c) in AzoPEG solution (same irradiation conditions) with swimmers. Сoncentric rings with the center at the laser point and a difference in thickness of 20 μm are shown for clarity.


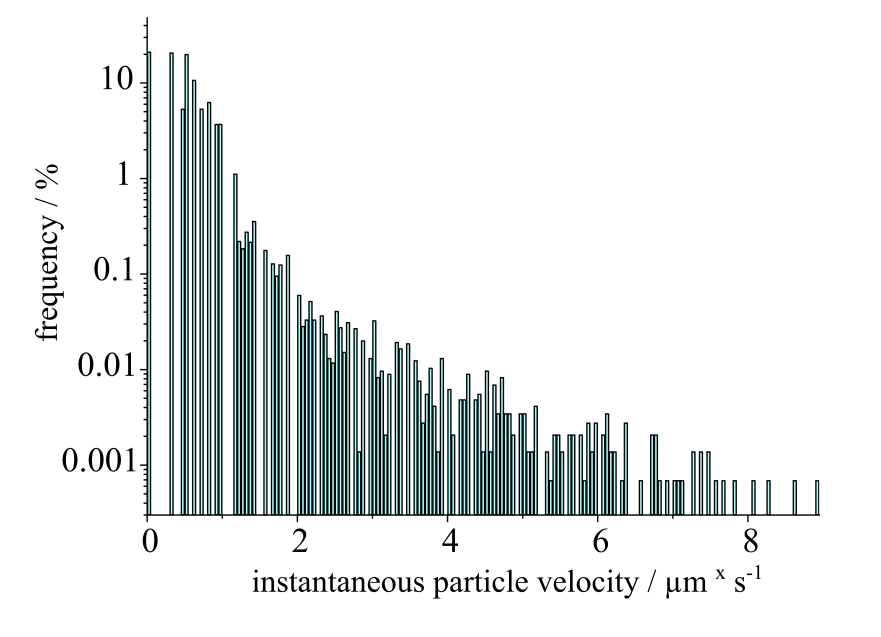


**Figure S12**. Distribution of the instantaneous (over 1s) particle velocity during irradiation with blue laser (*λ* = 488 nm, *P* =86 µW), in 150µM AzoPEG solution with swimmers at T = 30°C. Frequency was normalized by the total numbers of instant trajectories over 1 s, N=146500 .


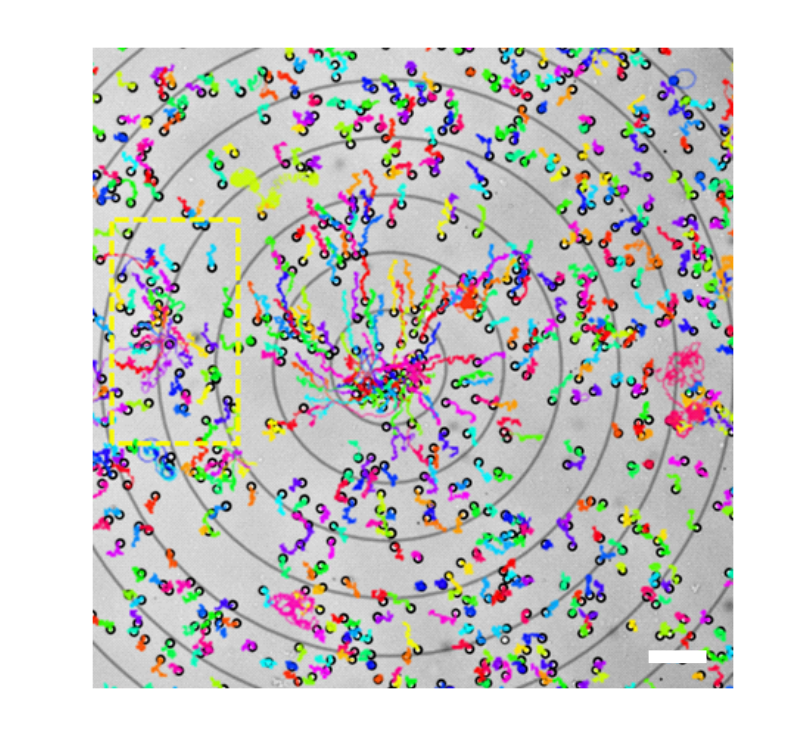


**Figure S13**. Trajectories of tracer particles in AzoPEG solution with *P. putida* swimmers (c = 150µM, 30°C, *λ* = 488 nm, *P* =86 µW). Сoncentric rings with the center at the laser point and a difference in thickness of 40 μm are shown for clarity. The yellow rectangle marks the area where spontaneous agglomeration of particles is observed. Long entangled trajectories indicate chaotic particle movement associated with the influence of bacteria. Scale bar is 40 µm.

**REFERENCES**

1. Billamboz, M. *et al.* Micellar catalysis using a photochromic surfactant: Application to the pd-catalyzed tsuji-trost reaction in water. *J. Org. Chem.* **79**, 493–500 (2014).

2. Muraveva, V. *et al.* Interplay of diffusio- and thermo-osmotic flows generated by single light stimulus. *Appl. Phys. Lett.* **120**, 231905 (2022).

3. Ortner, P., Umlandt, M., Lomadze, N., Santer, S. & Bekir, M. Artifact Correction of Light Induced Detuning in QCM-D Experiments. *Anal. Chem.* **95**, 15645–15655 (2023).
