# Supplementary material for: Manipulation of artificial and living small objects by light driven diffusioosmotic flow
Source: Sci Rep. 2024 Aug 7;14:18342. doi: 10.1038/s41598-024-69001-6 (PMC11306628; doi:10.1038/s41598-024-69001-6)
Supplement: Supplementary file 1 — Supplementary Information. [file 41598_2024_69001_MOESM1_ESM.zip › legend to Video S3.docx]

**Video S3**. Moving outwards blue laser beam irradiation (λ =488nm, P= 39 µW) colloids in the *cis*-isomers enriched AzoPEG solution (c=150 µM, T=25°C). To keep the *cis*-isomer concentration constant, irradiation with UV LED (λ =365nm, I= 3.6 mW/cm^2^*)* over the whole sample is carrying on. The corresponding time is depicted on the video (hours:minutes:seconds). Scale bar is 40 µm.
