# Supplementary material for: Manipulation of artificial and living small objects by light driven diffusioosmotic flow
Source: Sci Rep. 2024 Aug 7;14:18342. doi: 10.1038/s41598-024-69001-6 (PMC11306628; doi:10.1038/s41598-024-69001-6)
Supplement: Supplementary file 1 — Supplementary Information. [file 41598_2024_69001_MOESM1_ESM.zip › legend to Video S4.docx]

**Video S4**. Collection of colloids (d=5 μm) by UV laser irradiation (λ=375 nm, P=0.7 µW) under heating. Temperature keeping constant by a heating stage, T = 40° C. AzoPEG surfactant concentration c=75 µM. The corresponding time is depicted on the video (hours:minutes:seconds). Scale bar is 40 µm.
