# Supplementary material for: Manipulation of artificial and living small objects by light driven diffusioosmotic flow
Source: Sci Rep. 2024 Aug 7;14:18342. doi: 10.1038/s41598-024-69001-6 (PMC11306628; doi:10.1038/s41598-024-69001-6)
Supplement: Supplementary file 1 — Supplementary Information. [file 41598_2024_69001_MOESM1_ESM.zip › legend to Video S5.docx]

**Video S5**. Worm-like aggregates growing in the AzoPEG surfactant solution (c=150 µM) pre-irradiated with global UV-light (λ=365nm, I=8 mW/cm^2^) and during irradiation with focused high intensity blue light (λ=488 nm, P = 180 µW). The corresponding time is depicted on the video (hours:minutes:seconds). Scale bar is 40 µm.
