# Supplementary material for: Manipulation of artificial and living small objects by light driven diffusioosmotic flow
Source: Sci Rep. 2024 Aug 7;14:18342. doi: 10.1038/s41598-024-69001-6 (PMC11306628; doi:10.1038/s41598-024-69001-6)
Supplement: Supplementary file 1 — Supplementary Information. [file 41598_2024_69001_MOESM1_ESM.zip › legend to Video S6.docx]

**Video S6**. Bacteria *P. putida* swimming near the surface in motility buffer (control media) recorded 20 frames per second. Time t =12 min after dilution concentrate cells suspension by motility buffer. The corresponding time is depicted on the video (hours:minutes:seconds). Scale bar is 50 µm.
