# Supplementary material for: Manipulation of artificial and living small objects by light driven diffusioosmotic flow
Source: Sci Rep. 2024 Aug 7;14:18342. doi: 10.1038/s41598-024-69001-6 (PMC11306628; doi:10.1038/s41598-024-69001-6)
Supplement: Supplementary file 1 — Supplementary Information. [file 41598_2024_69001_MOESM1_ESM.zip › legend to Video S8.docx]

**Video S8**. Collected by LDDO flow silica particles (d = 5µm) during irradiation with blue laser (λ=488 nm, P=10 µW) at t_1_=2 minutes and t_2_=10 minutes of light exposure. Holes in the collection group occur due to active cell movement, which pushes particles away and destroy colloidal ordering. The corresponding time is depicted on the video (hours: minutes: seconds). Scale bar is 50 µm.
