# Supplementary material for: Manipulation of artificial and living small objects by light driven diffusioosmotic flow
Source: Sci Rep. 2024 Aug 7;14:18342. doi: 10.1038/s41598-024-69001-6 (PMC11306628; doi:10.1038/s41598-024-69001-6)
Supplement: Supplementary file 1 — Supplementary Information. [file 41598_2024_69001_MOESM1_ESM.zip › legend to Video S2.docx]

**Video S2**. Collection of tracers’ colloids (d=5µm) dispersed in AzoPEG (c=150 µM) aqueous solution by blue laser irradiation (λ=488 nm, P=9.3 µW). The corresponding irradiation time is depicted on the video (hours: minutes: seconds). Scale bar is 40 µm.
